# Antigen-specific T_H_17 cells offset the age-related decline in durable T cell immunity

**DOI:** 10.1126/sciadv.aea7131

**Published:** 2026-02-06

**Authors:** Ines Sturmlechner, Abhinav Jain, Jingjing Jiang, Hirohisa Okuyama, Yunmei Mu, Maryam Own, Cornelia M. Weyand, Jörg J. Goronzy

**Affiliations:** ^1^ Department of Immunology, Mayo Clinic, Rochester, MN 55905, USA.; ^2^ Robert and Arlene Kogod Center on Aging, Mayo Clinic, Rochester, MN 55905, USA.; ^3^ Department of Medicine, Division of Rheumatology, Mayo Clinic, Rochester, MN 55905, USA.

## Abstract

Older adults are susceptible to infections in part due to waning of immune memory. To uncover mechanisms of a long-lasting immune memory, we contrasted varicella zoster virus antigen–specific memory T cell responses in adults vaccinated at young (<20 years) or older age (>50 years) with a live-attenuated vaccine conferring durable protection only when given at young age or with an adjuvanted component vaccine eliciting long-lasting immunity in older adults. Unlike VZV-specific CD4^+^ T cells, CD8^+^ T cells exhibited profound age-sensitive changes including memory subset shifts, reduced T cell receptor diversity, and loss of stem-like features. Vaccination of older adults with the adjuvanted vaccine did not restore CD8^+^ defects but selectively enhanced T helper 17 (T_H_17) CD4^+^ T cells and prevented their conversion into regulatory T cells, likely through lipid metabolic regulation. Thus, durable vaccine efficacy with aging relies on antigen-specific T_H_17 cells that compensate for CD8^+^ T cell defects.

## Teaser

Aging impairs the CD8^+^ T cell VZV vaccine response, while effective VZV vaccination functions through inducing T_H_17 CD4^+^ T cells.

## INTRODUCTION

Vaccination is one of the most successful interventions in modern medicine, saving countless lives and improving public health globally. Childhood vaccinations have been transformative; however, infectious diseases remain a major cause of morbidity and mortality in older (O) adults when vaccinations are less efficacious. Numerous studies have tried to define the underlying age-related defects in the immune system, broadly referred to as immunosenescence (*1*–*3*). Successful vaccinations rely on the induction of immunologic memory that is mediated by B and T lymphocytes and plasma cells and typically manifest as the presence of antibodies in sufficient concentrations to neutralize the pathogen as well as the rapid T effector cell generation when the respective pathogen is encountered later in life (*4*–*6*).

Most previous studies on vaccine responses in older adults have focused on memory generation during the first months after vaccinations by describing the increase in antibody titers and the frequencies of antigen-specific T follicular helper cells (T_FH_ cells) or cytokine-producing effector T cells as readout systems (*7*, *8*). Several immune defects in older age have been proposed, but many of the age-associated changes are relatively small and only incompletely explain the reduced clinical efficacy of vaccines in older adults. The responding T cell population is reduced in numbers, and their ability to respond to T cell receptor (TCR) stimulation is blunted. DNA damage response pathways are increasingly compromised with aging (*9*, *10*), which may exacerbate genomic stress and impair the rapid expansion of T cells that T cell immunity highly relies on. While proliferating, T cells undergo fate decisions and differentiate into multiple phenotypic and functional T cell subsets. Fate decisions in older adults favor short-lived effector over longer-lived memory cells or follicular helper cells (*11*). Germinal center reactions involving the communication between B cells and T_FH_ cells are impaired, resulting in reduced generation of memory B cells and antibody-secreting, long-lived plasma cells (*12*). Fate trajectories are dynamic and develop over several months after vaccination (*13*, *14*), a process that appears to be particularly vulnerable in the aging host with a failure to develop stem-like memory T cells.

A key determinant of successful vaccination is the durability of immune memory. Very few studies have examined the mechanisms determining long-term durability over years or decades even in young (Y) adults. Memory durability appears to differ depending on the vaccine type. A vaccine that induces substantially durable immune memory is the live-attenuated yellow fever virus vaccine that confers life-long immunity (*15*). An example of differentially waning immunity was noted with the transition from the whole-cell pertussis vaccine to the acellular vaccine (*16*, *17*). The change in vaccination was associated with the resurgence of *Bordetella pertussis* infection among adolescents. The acellular vaccine induced mainly T helper 2 (T_H_2) immune responses, while the whole-cell vaccine induced mainly T_H_1 and T_H_17 responses that may account for superior and sustained protection. Similarly, the live-attenuated *Salmonella typhi* vaccines provide a longer duration of protection than the polysaccharide vaccine, which may be due to a different balance between effector memory T cells and regulatory T cells (*18*, *19*).

Here, we used varicella zoster virus (VZV) vaccination as a tool to uncover mechanisms of T cell memory durability depending on age and vaccine type. VZV typically causes chickenpox within the first few years of life while establishing latency. Given that protection from VZV reactivation is mediated by T cells and not antibodies (*20*), we focused our analysis on T cells. Starting at about the age of 50 years, reactivation of the virus presenting as shingles is increasingly observed, culminating to 50% of older adults being affected by the age of 80 years (*21*). In 1995, the childhood VZV vaccine Varivax, a live-attenuated virus, was licensed in the US (*22*). Varivax requires a booster but protects from chickenpox and, so far, prevents shingles (*23*, *24*). The first vaccine used to protect older adults from shingles was Zostavax (Z), which uses the same virus strain as the childhood vaccination, however, at a ~14 times higher viral dose. As opposed to the highly effective and durable response in children and young adults to the VZV vaccine, the effect of Zostavax is relatively short-lived with protection rates waning to ~40% at 3 to 5 years postvaccination (*25*, *26*). In 2016, an adjuvanted VZV component vaccine was Food and Drug Administration approved, which is highly effective in preventing shingles even in >70-year-old adults (*27*). Shingrix (S) consists of a recombinant VZV glycoprotein [glycoprotein E (gE)] antigen and the AS01_B_ adjuvant system and induces a highly protective immune memory in older adults with little waning within 10 years postvaccination (*28*), a time window in which Zostavax protection has completely disappeared.

To pinpoint the molecular identity of T cell durability and efficacy, we leveraged the unique VZV vaccine landscape in the United States in 2021 to 2023 shortly after the introduction of Shingrix and discontinuation of Zostavax. This allowed us to recruit Shingrix and Zostavax vaccine recipients 3+ years after vaccination to directly compare the two contrasting vaccine T cell memories in older adults. In addition, we recruited younger adults having received Varivax, the same vaccine strain as Zostavax, in their childhood. We compared peripheral T cells specific for VZV gE, the same antigen shared by all three vaccines, which should allow for a more robust assessment of aging trajectories (*29*). Using trimodal single-cell sequencing, we found that VZV gE–specific CD4^+^ and CD8^+^ T cells greatly differ in their susceptibility to age. Antigen-specific CD4^+^ T memory cells were largely stable in older Zostavax vaccinees, except for a loss in the expression of interferon-related genes, however, without evidence for cellular senescence or exhaustion. In contrast, antigen-specific CD8^+^ T cells had developed into end-differentiated, effector-like cells and were contracted in TCR diversity, which may account for the loss in protective function. The more effective Shingrix vaccination did not restore these CD8^+^ T cell defects in older adults but selectively improved the functionality of VZV gE–specific CD4^+^ T_H_17 cells and prevented their degeneration into cells expressing regulatory T (T_reg_)–related genes. Collectively, our data indicate that effective vaccine-induced protection in older adults can be supported by the generation of a durable, antigen-specific CD4^+^ T_H_17 population.

## RESULTS

### Influence of age on frequencies and heterogeneity of VZV-specific memory T cells

**Fig. 1.**
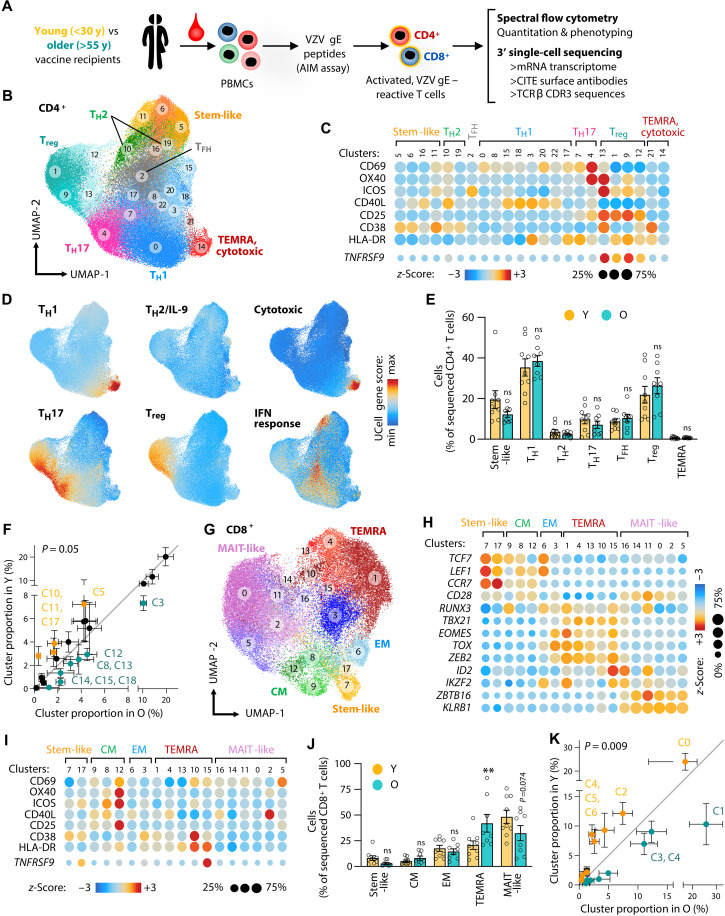
VZV gE–specific CD8^+^ T cells are highly age-sensitive, while CD4^+^ memory T cells are resilient. (**A**) Schematic of experimental design. y, years. (**B**) UMAP of VZV gE–reactive CD4^+^ T cells depicting distinct T cell clusters. (**C**) Dot plot heatmap of activation markers of CITE protein (top) or gene expression (bottom) showing nonuniform activation marker expression across CD4^+^ T cell clusters. (**D**) UCell gene score analysis of T helper signatures to annotate clusters to conventional T cell subsets. (**E** and **F**) Relative subset (E) or cluster (F) frequencies of CD4^+^ VZV gE–reactive T cells comparing young (Y) and older (O) vaccine recipients. Statistical analysis was performed by comparing probability vectors and permutation test (F). (**G**) UMAP of VZV gE–reactive CD8^+^ T cells. (**H**) Dot plot heatmap for VZV gE–responsive CD8^+^ T cells showing the gene expression of classical T cell subset markers. (**I**) Dot plot heatmap of activation markers of CITE protein (top) or gene expression (bottom) showing nonuniform activation marker utilization across CD8^+^ T cell clusters. (**J** and **K**) Relative subset (J) or cluster (K) frequencies of CD8^+^ VZV gE–reactive T cells in Y and O vaccine recipients. Data show the means ± SEM (E, F, J, and K). All data points represent distinct biological replicates. Data were compared by a two-way ANOVA with Šídák’s multiple comparisons test (E and J). **P < 0.01. ns, not significant.

Vaccination with the live-attenuated VZV strain in children (Varivax) is protective for >20 years, while the same vaccine strain in 50+-year-old adults (Zostavax) only induces partial and rapidly waning protection. To identify signatures in antigen-specific T cells that may account for the difference in protection and memory durability (Fig. 1A), we recruited individuals who had received either the childhood or adulthood vaccine. Postvaccination intervals were chosen for young adults (14 ± 1.7 years) and older adults (6 ± 0.8 years) after their last VZV vaccine dose. At the time of peripheral blood collection, these individuals were 24.3 ± 2.9 years (young adults) and 75.5 ± 7.3 years old (older adults) (tables S1 and S2). We optimized activation-induced marker (AIM) assays (*30*) to comprehensively identify and collect VZV antigen–specific CD4^+^ and CD8^+^ T cells. Culture conditions were chosen to optimize the expression of activation markers in peptide versus dimethyl sulfoxide (DMSO) solvent control cultures and to identify the maximal number of antigen-specific T cells before cells divided ex vivo (fig. S1, A to E). For our AIM assays, we chose 42 hours after stimulation of peripheral blood mononuclear cells (PBMCs) with overlapping peptides of VZV gE as the time point and CD69 and CD137 as the most inclusive activation markers for both CD4^+^ and CD8^+^ T cells as markers for fluorescence-activated cell sorting (FACS)–mediated cell collection.

We found that net frequencies of VZV gE–reactive CD4^+^ or CD8^+^ T cells did not differ in older VZV vaccine recipients compared to younger vaccinees (fig. S1F) despite the lower rate of shingles protection with age. To probe for qualitative changes in VZV gE–specific T cells, we subjected cells to single-cell sequencing with three modalities: 3′ RNA expression, surface proteome via 40 CITE (cellular indexing of transcriptomes and epitopes by sequencing) antibodies, and *TRB* CDR3 sequences.

We profiled VZV gE–responsive CD4^+^ and CD8^+^ T cells from nine young and eight older live-attenuated VZV vaccine recipients and obtained 43,593 CD4^+^ and 19,350 CD8^+^ VZV gE–reactive T cells (table S3). CD4^+^ T cells were clustered into 23 subtypes as visualized by uniform manifold approximation and projection (UMAP; Fig. 1B). Clustering resolution was chosen such that clusters corresponded to well-defined conventional subsets. Almost all clusters included one to several activation markers in addition to CD69 (Fig. 1C and fig. S2A). Frequently, the expression of these activation markers was cluster-specific, validating our approach to use broad markers to capture cells for sequencing and use bioinformatic analyses to assess more specific combinations of activation markers. Subset annotation was initially performed using transcriptional signature gene scores (Fig. 1D). Assessment of classical lineage-distinguishing cell surface molecules and the expression of key transcription factors, cytokines, and effector molecules confirmed the assignments (fig. S2, B to D). The VZV gE–specific CD4^+^ T cell response exhibited marked cell heterogeneity and included subsets with features of stem-like, T_H_1, T_H_17, T_reg_, T_H_2, T_FH_, and TEMRA (terminal effector memory expressing CD45RA) cells. The subsets T_H_1 (cluster, C0), T_reg_ (C1), and T_H_17 (C4) were dominating in the VZV gE response, which we confirmed by functional studies. To this end, we collected VZV gE–reactive CD4^+^ T cells by FACS on the basis of the single-cell sequencing–informed cell surface expression of CCR4, CCR6, IL-7R (interleukin-17 receptor), CD26, and TIGIT (T cell immunoreceptor with Ig and ITIM domains) that distinguished these subsets (fig. S3A). Purified cells were recultured, polyclonally stimulated with αCD3/αCD28 antibodies, and profiled for cytokine secretion, cell expansion, and survival (fig. S3, B to E). C1 (T_reg_) cells were predominantly FOXP3^+^ and IL-7R^lo^, did not proliferate under these conditions, and were more prone to undergo apoptosis. In contrast, C0 (T_H_1) and C4 (T_H_17) cells showed high expansion potential, survival, and differentially secreted high levels of cytokines including interferon-γ (IFN-γ), tumor necrosis factor–α (TNFα), and IL-17A.

To determine whether the CD4^+^ T cell subset heterogeneity in the VZV gE response differed between young and older vaccine recipients, we compared the proportion of conventional subset and examined the distribution of antigen-specific CD4^+^ T cells in the high-dimensional clustering data. We examined the distribution of cluster frequencies by comparing probability vectors and performing linear regression analyses. We found a trend towards significantly different subset distributions with age (*P* = 0.05; Fig. 1, E and F) with several individual clusters marginally dominating in young (orange font such as C5) or older vaccine recipients (teal font such as C3). These data suggest that the VZV gE response of CD4^+^ T cells and their phenotypic heterogeneity are largely stable with age.

### CD8^+^ T cells specific for VZV gE are highly age-sensitive

Contrary to VZV gE–responsive CD4^+^ memory T cells, the antigen-specific CD8^+^ T cell response was subjected to extensive changes with age. Single-cell clustering of VZV gE–responsive CD8^+^ T cells yielded 18 subsets (Fig. 1G), differing in their profile of cell surface lineage molecules, the expression of activation markers, lineage-determining transcription factors, and effector molecules (Fig. 1, H and I, and fig. S4, A and B). The CD8^+^ response was dominated by TEMRA and mucosal-associated invariant T (MAIT)–like cells, the latter showing a bias in TRAV (TCRα variable)-TRAJ (TCRα junction) usage characteristic of this subset (fig. S4C). Young and older vaccinated adults differed substantially and significantly (*P* = 0.009) in their preferred CD8^+^ cluster and cell type distributions (Fig. 1, J and K). Specifically, end-differentiated TEMRA subsets significantly increased with age, while stem- and MAIT-like cells tended to decrease.

**Fig. 2.**
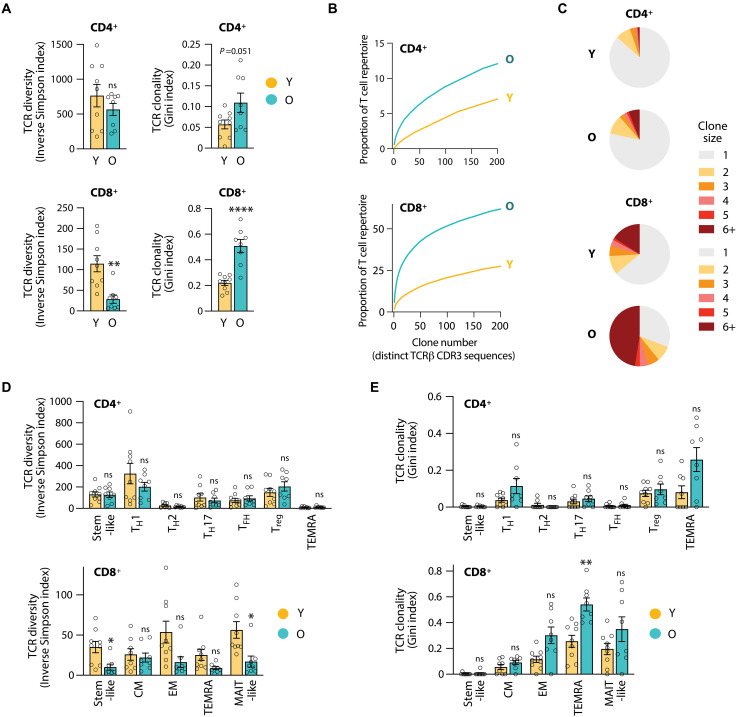
VZV gE–reactive CD4^+^ T cells maintain diversity with age while the repertoire of antigen-specific CD8^+^ T cells contracts. (**A**) TCR diversity and clonality based on single-cell TCRβ CDR3 sequences in VZV gE–reactive CD4^+^ and CD8^+^ T cells of Y or O vaccine recipients. (**B**) Cumulative TCR plots showing the TCRs ranked by the clone number in descending clone sizes versus the space they occupy. (**C**) Pie chart of clonal expansion with the size of the pie slices showing the proportion of clones of the indicated clone size. (**D** and **E**) TCR diversity index (D) and TCR clonality index (E) across T cell subsets of VZV gE–reactive CD4^+^ and CD8^+^ T cells in Y and O vaccine recipients. Data show the means ± SEM (A, D, and E). All data points represent distinct biological replicates. Data were compared by two-tailed, unpaired *t* tests (A) or a two-way ANOVA with Šídák’s multiple comparisons test (D and E). *P < 0.05, **P < 0.01, and ****P < 0.0001.

The sensitivity of CD8^+^ T cells to age was not restricted to phenotypic heterogeneity but also involved their TCR repertoire composition. While the VZV gE CD4^+^ T cell response was highly diverse with largely unchanged TCR diversity indexes and only a trend to increased clonality in older vaccine recipients, CD8^+^ T cells were again subject to considerable age-related changes (Fig. 2A). TCRβ chain diversity in VZV gE–specific CD8^+^ T cells was nearly 10-fold lower than in CD4^+^ T cells even in young adults and further markedly decreased in older vaccine recipients. Plotting the numbers of distinct VZV gE–reactive TCRβ chains ordered by decreasing clonal sizes versus the cumulative space they occupy illustrated the high degree of diversity of antigen-specific CD4^+^ T cells (Fig. 2, B and C). Again, diversity was strongly contracted for CD8^+^ T cells but was only mildly affected for CD4^+^ T cells of older individuals.

The loss in CD8^+^ T cell diversity affected all subsets, except the infrequent central memory cells, reaching significance for stem- and MAIT-like T cells (Fig. 2D). TCR clonality was increased in multiple subsets, predominantly in effector memory populations, and reached significance in the CD8^+^ TEMRA population of older adults (Fig. 2E). Diversity and clonality indices in CD4^+^ T cells were unchanged with advanced age. These data indicate that reduced protection is unlikely to be attributed to differences in the frequencies and repertoires of antigen-specific CD4^+^ T cells; rather, the age-associated contraction in CD8^+^ T cell diversity may increase the vulnerability of older adults.

### Increased interferon response in antigen-specific CD4^+^ T cells from young vaccine recipients

**Fig. 3.**
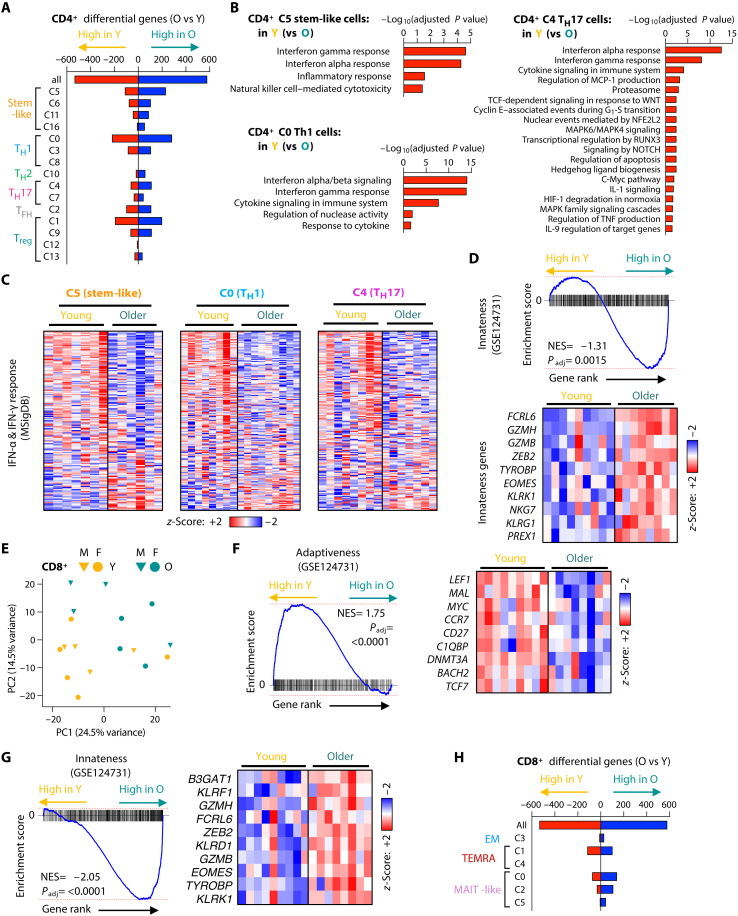
The type and extent of age-associated transcriptomic remodeling depend on the VZV gE–responsive T cell subset. (**A**) DEGs in each CD4^+^ single-cell RNA sequencing cluster as identified by pseudobulk differential expression in O versus Y vaccine recipients. Only clusters with ≥2 male and ≥2 female subjects per group are included. (**B**) Pathway enrichment for genes higher expressed in Y adults than in O adults in the indicated CD4^+^ clusters. (**C**) Gene expression heatmaps for gene sets “IFN-α response” and “IFN-γ response” (MSigDB) based on pseudobulk expression comparing O versus Y vaccine recipients for three CD4^+^ T cell clusters (C5, C0, and C4). (**D**) GSEA comparing pseudobulk gene expression in CD4^+^ T cells from O versus Y vaccine recipients with the lymphocyte “innateness” gene set (GSE124731). Pseudobulk gene expression heatmap showing 10 genes from the “innateness” gene set. NES, normalized enrichment score. (**E**) Principal component (PC) analysis of pseudobulk gene expression data on CD8^+^ VZV gE–specific T cells for each vaccine recipient (M, male; F, female). (**F** and **G**) GSEA and heatmaps for CD8^+^ VZV gE–specific T cells from O versus Y vaccine recipients. (**H**) Differential gene expression in CD8^+^ VZV gE–specific T cells from O versus Y adults shown for total CD8^+^ T cells and indicated clusters.

To probe for molecular signatures contributing to the age-related defect in T cell memory against VZV, we next assessed the gene expression profiles of memory T cell clusters. Dimension reduction of complex gene expression data using principal components analysis (PCA) showed a small shift in PC1 and PC2 of VZV gE–reactive CD4^+^ T cells from young and older vaccine recipients (fig. S5A). Pseudobulk differential expression analyses identified 579 up-regulated and 535 down-regulated genes in older adults. Subset clusters were unevenly affected with most differentially expressed genes (DEGs) being found in T_H_1 (C0), T_reg_ (C1), and stem-like (C5) cells (Fig. 3A). Analysis of C1 T_reg_ DEGs did not identify enriched pathways. The stem-like C5 cluster lost self-renewing, stem-like–related transcription factors including *TCF4*, *LEF1*, and *FOXO1* in older adults (fig. S5B). In addition, C5 DEGs in young adults showed an enrichment for type I and II interferon responses, followed by increased natural killer cell (NK cell)–related features (Fig. 3B). Conversely, C5 DEGs up-regulated with age were enriched for pathways of translational and metabolic activity (fig. S5C). An even larger enrichment for interferon responses at young age was seen in T_H_1 C0 and T_H_17 C4 cells (Fig. 3, B and C), suggesting that blunting of the interferon response with age is a common signature across multiple antigen-specific T cell subsets. This defect was not a sign of exhaustion of VZV gE–reactive CD4^+^ T cells as assessed by gene set enrichment analysis (GSEA) (fig. S5, D and E). Moreover, these T cells did not exhibit evidence of cellular senescence (fig. S5, F and G). Unexpectedly, CD4^+^ T cells of older vaccine recipients had gained innate-like lymphocyte features, with their gene expression correlating with a gene set that had recently been described as designating the trajectory from CD4^+^ over CD8^+^, MAIT, invariant NK T, and γδ T cells to NK cells (*31*) and that is also enriched in Epstein Barr virus–specific CD8^+^ T cells of older adults (Fig. 3D) (*29*). These data suggest that although largely stable in their numbers, TCR diversity, and T cell subset phenotype, VZV gE–specific memory CD4^+^ T cells adapt their intrinsic response pathways with aging to shift toward potent effector, innate lymphocyte properties at the expense of interferon-response genes.

### VZV gE–specific CD8^+^ T cells shift from adaptive to innate gene signatures with age

VZV gE–specific CD8^+^ T cells showed even more marked transcriptional changes with age, resulting in a clear separation of the gene expression profiles of young and older vaccine recipients by PCA (Fig. 3E). GSEA identified a highly significant enrichment for stemness, adaptive immunity features in young vaccine recipients, and innate lymphocyte immunity in older vaccine recipients (Fig. 3, F and G). In contrast, similar to the CD4^+^ T cell compartment, evidence for exhaustion and cellular senescence modules were low or absent (fig. S6, A to D). A total of 1509 DEGs were identified in the pseudobulk gene expression comparison of VZV gE–specific CD8^+^ T cells from young and older vaccine recipients. These differences in gene expression were largely attributed to the shifts in subset distributions as described in Fig. 1. Only a small number of DEGs were identified when pseudobulk analyses for each cluster were compared (Fig. 3H). Pathway analyses of these cluster-specific DEGs showed up-regulation of translational processes in older MAIT-like cells and down-regulation of signaling pathways in older TEMRA cells (fig. S6, E and F). Collectively, these analyses indicate a shift of VZV gE–specific CD8^+^ T cells from self-renewing, stem-like T cell subsets to potent effector, innate-like subsets with age.

### VZV gE–specific T_H_17 response in older recipients of adjuvanted vaccines

**Fig. 4.**
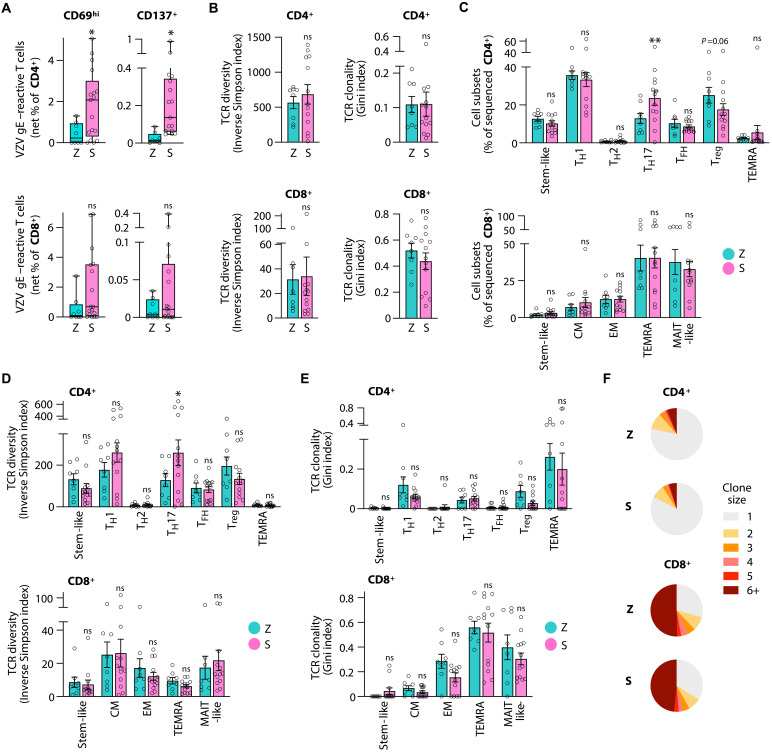
Shingrix vaccination induces an abundant, sustained, and T_H_17-rich T cell response. (**A**) Frequencies of VZV gE–reactive CD4^+^ and CD8^+^ T cells from older adults who were vaccinated with the Zostavax (Z) or Shingrix (S) vaccine. VZV gE–reactive T cells were identified as CD69^hi^ or CD137^+^ by flow cytometry; results are shown as background control–subtracted (net) frequencies. (**B**) TCR diversity and clonality indices based on single-cell TCRβ CDR3 sequences in VZV gE–reactive CD4^+^ and CD8^+^ T cells from Z and S vaccine recipients. (**C**) Distribution of CD4^+^ and CD8^+^ VZV gE–reactive T cells from Z or S vaccine recipients across T cell subsets derived from the single-cell RNA sequencing cluster analysis shown in fig. S8. (**D** and **E**) TCR diversity index (D) and clonality index (E) of CD4^+^ and CD8^+^ subsets from Z and S recipients. (**F**) Pie chart of clonal expansion with the size of the pie slices showing the proportion of clones of the indicated clone size. Data show the median (A) or means ± SEM (B to E). All data points represent distinct biological replicates. Data were compared by Mann-Whitney tests (A); two-tailed, unpaired *t* tests (B); and a two-way ANOVA with Šídák’s multiple comparisons test (C to E). *P < 0.05 and **P < 0.01.

An adjuvanted VZV gE component vaccine (Shingrix) that was licensed in 2017 in the US provides efficient and long-term protection in older adults as opposed to the live-attenuated virus vaccine (Zostavax). To investigate how Shingrix elicits this exceptional protection despite age, we sought out to identify molecular determinants in T cells that may explain the higher durability of immunity. We recruited 13 individuals for single-cell sequencing who had been vaccinated with Shingrix shortly after the introduction of the vaccine (ranging from 3 to 5 years ago). At the time of peripheral blood collection, Shingrix recipients were 69.5 ± 7.3 years old (referred to as the Shingrix group, S), comparable to the described Zostavax recipients (as of now referred to as the Zostavax group, Z) (tables S1 and S2). We collected VZV gE–reactive T cells from Shingrix recipients by peptide stimulation of donor PBMCs and analyzed them by spectral flow cytometry and single-cell sequencing (retaining 34,119 CD4^+^ and 9989 CD8^+^ sequenced VZV gE–reactive T cells). In line with a previous report (*32*), the frequencies of VZV gE–specific CD4^+^ and CD8^+^ T cells were higher after Shingrix vaccination, irrespective of which activation marker was used (Fig. 4A and fig. S7, A and B). In contrast, responses to an unrelated superantigen, SEB (staphylococcal enterotoxin B), were not different between the two groups (fig. S7C), suggesting similar general immunologic fitness of both vaccine recipient groups. Increased antigen-specific frequencies were not associated with a difference in TCR diversity or clonality for neither VZV gE–specific CD4^+^ nor CD8^+^ T cells as concluded from Inverse Simpson and Gini indices (Fig. 4B). Similarly, cumulative TCRβ-chain frequency plots showed no or minor differences with slightly higher diversity and reduced clonality for VZV gE–specific CD4^+^ T cells of Shingrix recipients (fig. S7D).

When determining whether the longer durability of protective immune memory was associated with T cell subset shifts (fig. S8, A to F), we did not find any significant differences in probability vectors describing global subset distributions, neither for VZV gE–specific CD4^+^ nor CD8^+^ T cells (fig. S7G). However, we noted a significant increase in CD4^+^ T_H_17 cells as well as a trend toward decreased T_reg_ cells in Shingrix recipients (Fig. 4C). Increased T_H_17 cell numbers were associated with higher TCR diversity (Fig. 4D) without affecting clonality (Fig. 4, E and F), possibly indicating the recruitment of new T cell specificities upon vaccination into this compartment (*33*). VZV gE–specific CD8^+^ T cells did not differ between Shingrix and Zostavax recipients, suggesting that Shingrix vaccination did not simply rejuvenate the VZV gE response to a status seen in young vaccine recipients (Figs. 1 and 2). Rather, Shingrix vaccination appeared to mobilize the CD4^+^ T cell response to confer vaccine protection.

### Plasticity in VZV gE–responsive T_H_17 cells in Shingrix and Zostavax recipients

**Fig. 5.**
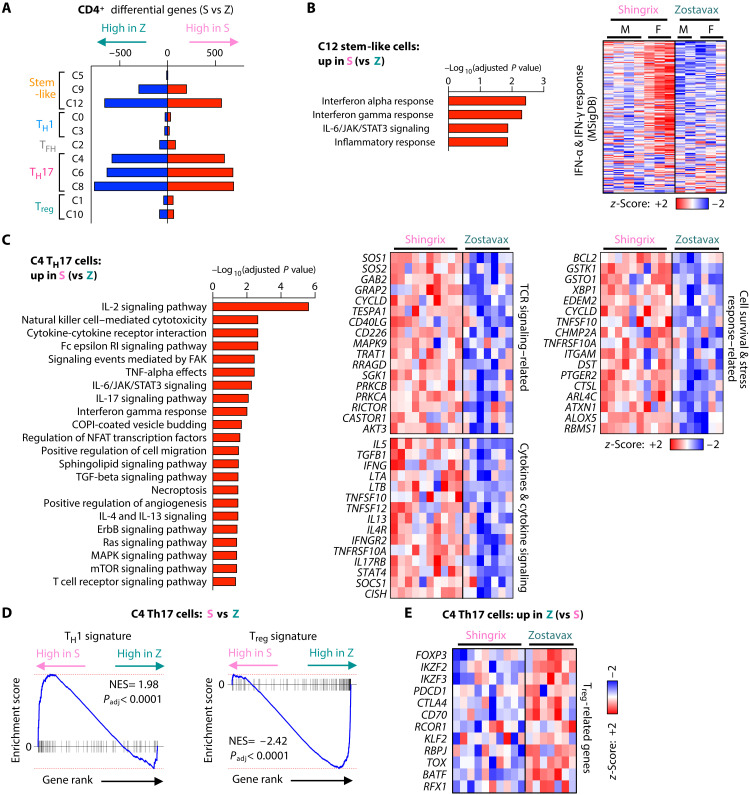
Gene expression in T_H_17 cells indicates increased functionality in Shingrix vaccinees. (**A**) DEGs in each CD4^+^ T cell single-cell RNA sequencing cluster as identified by pseudobulk differential gene expression between S and Z vaccine recipients. Only clusters with a minimum sample representation of two male and two female participants per group are included. (**B** and **C**) Pathway enrichment of C12 (B) and C4 (C) DEGs higher expressed in S than in Z (left). Pseudobulk gene expression heatmaps of “IFN-α response” and “IFN-γ response” gene sets [(B), right] and selected DEGs of indicated pathways [(C), right]. (**D**) GSEA of C4 pseudobulk gene expression in Z versus S for concordance with T_H_1 and T_reg_ gene sets. (**E**) Gene expression heatmap of selected DEGs higher expressed in Z as compared to S.

VZV gE–specific CD4^+^ T_H_17 cells not only differed in frequencies but also showed diverging transcriptomes between Shingrix and Zostavax recipients. The PCA of the most variable transcripts showed a trend toward separation of Zostavax and Shingrix recipients for VZV gE–specific CD4^+^ T cells (fig. S9A) but not for CD8^+^ T cells (fig. S9B). CD4^+^ T cell clusters were unevenly affected by transcriptomic differences with many more DEGs (>1000) for clusters with T_H_17 and stem-like signatures (Fig. 5A). Enriched pathways of Shingrix-induced DEGs in stem-like cluster 12 included type I and II interferon–related genes (Fig. 5B), thereby reversing the age-associated decline in interferon response shown in Fig. 3. Notably, this higher expression was predominantly driven by female subjects. Although sample sizes are small, we further investigated whether sex-dependent differences exist in older VZV vaccine recipients. We found that the interferon response was also restored specifically in female Shingrix recipients in T_H_1 cluster 0 and T_H_17 cluster 4 (fig. S9C). However, memory subset heterogeneity did not differ in male and female vaccine recipients in VZV gE–specific CD4^+^ (fig. S9D) nor CD8^+^ T cells (fig. S9E). The serostatus for cytomegalovirus (CMV), another variable known to affect the immune status of individuals, had also only minor effects on the VZV memory subset heterogeneity (fig. S9F).

For the T_H_17 clusters C4, C6, and C8, Shingrix recipients showed the increased expression of genes indicative of superior functionality (Fig. 5C and fig. S10, A and B). In addition to interferon-related genes, TCR, and cytokine signaling, survival and stress-response pathways were enriched. Differential expression of these genes in the T_H_17 subsets included all recipients irrespective of their sex. The GSEA of these T_H_17 clusters C4, C6, and C8 showed a significant correlation with a T_H_1 gene signature in Shingrix recipients, suggesting a pro-inflammatory potential and, possibly, polyfunctionality (Fig. 5D and fig. S10, C and D). In contrast, DEGs with a higher expression in VZV gE–responsive T_H_17 cells of Zostavax recipients were indicative of T_reg_ traits with a higher expression of classical T_reg_ markers *FOXP3*, *IKZF2*, and *CTLA4*, as well as genes promoting T_reg_ stability and function, including *RCOR1* (*34*), *KLF2* (*35*), *RBPJ* (*36*), *TOX*, and *BATF* (Fig. 5, D and E) (*37*, *38*). T_reg_ genes were also lower and T_H_1 traits were higher in Shingrix T_H_17 cells when comparing them to T_H_17 cells of younger, Varivax vaccine recipients discussed in Fig. 1, suggesting predominant effects of the adjuvanted Shingrix vaccine compared to the live-attenuated virus vaccine independent of age (fig. S10E). Transcriptomic differences were largely reproducible when we focused on clonally expanded T_H_17 cells, a cell population highly enriched for VZV antigen specificity (fig. S10, F and G). DEGs comparing clonally expanded T_H_17 cells of Shingrix and Zostavax recipients significantly overlapped with our initial analysis encompassing all gE-activated T_H_17 cells. Consistently, genes elevated with Shingrix vaccination included proteins supporting T cell functionality, while those induced with Zostavax included typical T_reg_ genes like HELIOS (*IKZF2*) and *TIGIT*. These data suggest divergent phenotypic skewing of antigen-specific T_H_17 cells with Shingrix prioritizing pro-inflammatory, T_H_1-like features and Zostavax predisposing to immunosuppressive T_reg_ traits.

### Opposing differentiation trajectories of VZV gE–responsive T_H_17 cells

**Fig. 6.**
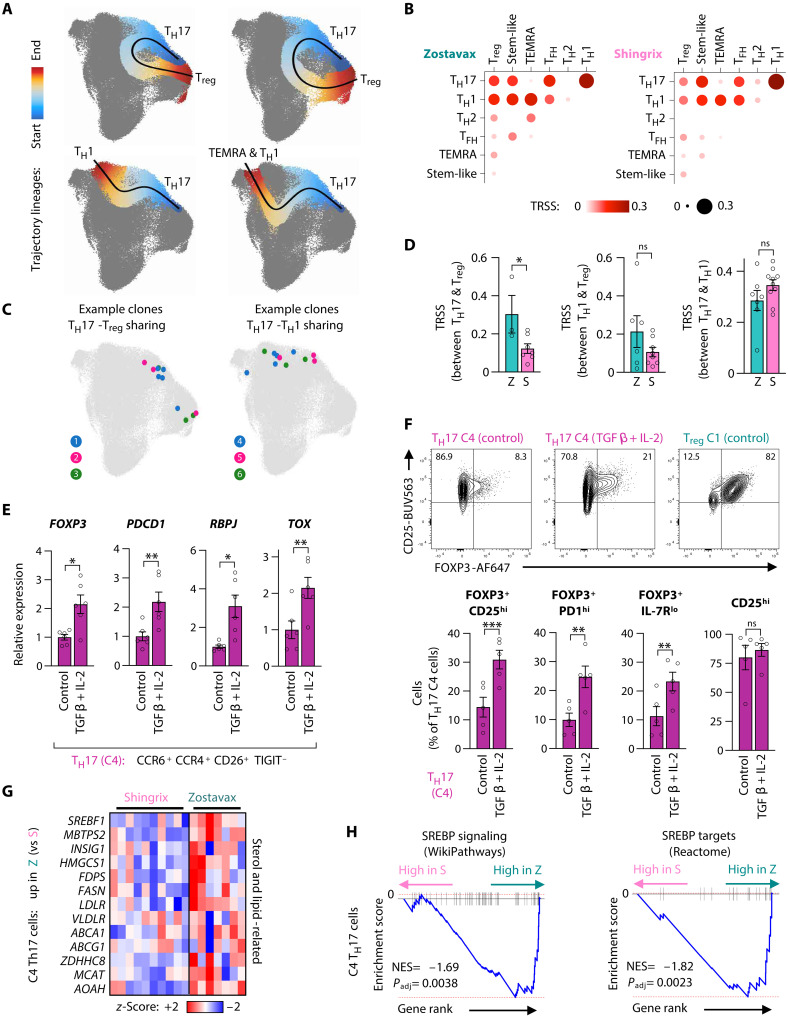
T_H_17 cells differentiate toward regulatory phenotypes. (**A**) Slingshot trajectory analysis projected on the UMAP space. T_H_17 cluster 4 was selected as the starting point to assess putative differentiation potentials. (**B**) Bubble plot of clonotype tracking across CD4^+^ VZV gE–reactive T cell subsets. TRSSs are shown as the color gradient and bubble size. (**C**) Representative TCR-identical clones with divergent transcriptional phenotypes found across different clusters. (**D**) TRSS per vaccine recipient showing the extent of TCR sharing between selected clusters. Only samples with at least 10 expanded clones in a given cluster combination were considered. (**E** and **F**) FACS-collected CD4^+^ memory T cells with a cluster 4 T_H_17 phenotype were stimulated with αCD3/αCD28 in the absence or presence of TGFβ (25 ng/ml) and IL-2 (500 U/ml) for 3 days (E) or 7 days (F). qPCR from three independent experiments (E) and FACS data from two independent experiments (F) are shown. Cluster 1 cells with a T_reg_ phenotype are shown as a comparison. (**G**) Gene expression heatmap of lipid metabolism–related DEGs in T_H_17 C4. (**H**) GSEA of SREBP (*SREBF1*) signaling and transcriptional target genes in T_H_17 C4 cells. Data show the means ± SEM (D to F). All data points represent distinct biological replicates. Data were compared by two-tailed, unpaired *t* tests (D) or two-tailed, paired *t* tests (E and F). *P < 0.05, **P < 0.01, and ***P < 0.001.

T_H_17 cells are capable of phenotypic and functional plasticity along the inflammatory and suppressive T cell spectrum including the differentiation into nonclassical T_H_1 cells (*39*). We therefore investigated the differentiation potential of VZV gE–specific T_H_17 cells using trajectory analyses on our single-cell sequencing data (Fig. 6A). Putative trajectories between T_H_17 C0 cells and either T_H_1 clusters or regulatory clusters suggested multipotent differentiation potentials of antigen-specific T_H_17 cells. We further analyzed TCR clonotype sharing across T cell subsets (*40*), which relies on cells containing the same TCR but divergent gene expression patterns and subset affiliations. TCR sharing was found between the T_H_17 and T_H_1 lineages as well as between T_H_17 and regulatory cells (Fig. 6, B and C). Zostavax recipients exhibited higher proportions of TCR sharing (~30%) between T_H_17 and regulatory cells as compared to ~10% in Shingrix recipients (Fig. 6, B to D). In contrast, T_H_17-T_H_1 sharing did not differ between vaccine groups. These data suggest that VZV gE–specific T_H_17 cells have the potential to misdifferentiate into regulatory cells, a process that is more enabled in Zostavax recipient cells.

To experimentally test whether cells with a T_H_17 (C4) phenotype can acquire features of regulatory cells, we FACS collected these cells and stimulated them polyclonally in the absence or presence of the classical T_reg_-inducing cytokine combination of transforming growth factor–β (TGFβ) and IL-2 for 3 days. Quantitative polymerase chain reaction analysis for the T_reg_ marker *FOXP3* as well as T_reg_-related T_H_17 signature genes *PDCD1*, *RBPJ*, and *TOX* in Zostavax recipients (fig. S11A; see also Fig. 5E) confirmed that T_H_17 cells can acquire T_reg_ traits (Fig. 6E). Flow cytometry analysis of these cells after 7 days corroborated the gain of a FOXP3^+^ T_reg_ phenotype in T_H_17 cells after treatment with TGFβ and IL-2 (Fig. 6F and fig. S11, B and C).

We investigated molecular pathways that may contribute to the differentiation fates of VZV gE–reactive T_H_17 cells toward regulatory features. Among the top up-regulated genes in Zostavax T_H_17 C4 cells, we noticed *SREBF1*, encoding sterol regulatory element–binding protein 1 (SREBP1), a master transcription factor of lipogenesis, and many of its associated enzymes and downstream targets including the SREBP-activating enzyme *MBTPS2* encoding S2P, *HMGCS1*, *FPS*, and *FASN* (Fig. 6G). GSEA on SREBP signaling and transcriptional target gene sets confirmed higher SREBP activity in T_H_17 cells of Zostavax individuals (Fig. 6H). Lipid metabolic pathways involving fatty acids and sterols, including those controlled by SREBP, have been intricately linked to T cell phenotypes and function (*41*), particularly promoting T_reg_ fitness and suppressiveness (*42*, *43*) as well as modulating pro-inflammatory or suppressive T_H_17 fates (*44*, *45*). In summary, we conclude that T_H_17 cells, particularly those being induced after Zostavax vaccination, can misdifferentiate toward regulatory phenotypes.

## DISCUSSION

The durability of immune memory is of critical importance in designing vaccination strategies and determines the need for booster vaccination. Waning of immune memory is of particular relevance to the older population where it contributes to the increased susceptibility to infections and a poorer vaccine response. In considering mechanisms of durability, it is important to note that memory cells are a dynamic population. Memory is not conferred by the longevity of individual cells but by a population of cells that individually are more short-lived (*46*). Memory T and B cells have limited lifespans, mostly considerably shorter than the duration of immunologic memory. The lifespan of a human memory T cell is 30 to 160 days, in contrast to the typical half-life of human T cell memory of 8 to 15 years (*47*–*50*). One exception is a small population of long-lived, stem-like memory cells that have a high degree of self-renewal and a half-life of ~9 years (*51*). Memory therefore reflects population averaging of a very diverse set of cells that are subject to selection pressure even in the absence of iterative or chronic antigenic stimulation.

Here, we used VZV vaccination as a model system to uncover determinants of an effective, durable T cell memory by comparing the adaptations of VZV-specific memory T cells many years after vaccination. Specifically, we examined to which extent cell lifespan, contraction in TCR diversity, transition between different functional states, and development of cellular senescence or exhaustion contribute to a lack in durable T cell memory. We find that age-associated changes comparing young and older adults vaccinated with a live-attenuated virus vaccine are much more prominent for antigen-specific CD8^+^ than CD4^+^ T cells. The adjuvanted component vaccine Shingrix induces superior durability without restoring these defects in CD8^+^ immunity but by inducing compensatory properties in CD4^+^ T cells. Moreover, neither VZV gE–responsive CD4^+^ nor CD8^+^ T cells acquire a transcriptional signature of cellular senescence or exhaustion. While we do not directly measure functional changes, our analyses of activation-induced transcriptome and CITE surface protein signatures should be a valid indicator of function. Our observations are consistent with a recent study in which antigen-specific CD8^+^ T cells in mice undergoing iterative viral infections have an infinite lifespan as long as there is a minimal interval of several weeks between stimulations (*52*).

An essential role in maintaining long-term memory, in particular for CD8^+^ T cells, has been attributed to a small population of stem-like memory T cells that shares features of naïve and memory cells and that is able to reconstitute the full spectrum of memory and effector T subpopulations (*51*). Transcription factors that are pivotal for preserving stemness include TCF1 and LEF1. A decline in TCF1 levels is a hallmark of the aging process in global naïve CD4^+^ T cells (*53*), and lower expression of TCF1 in CD8^+^ than CD4^+^ naïve T cells may contribute to their higher loss with aging. Frequencies of VZV gE-reactive stem-like cells tended to be reduced with age for both CD4^+^ and CD8^+^ T cells and DEGs in the stem-like subsets included several genes of functional importance to stemness, suggesting that this mechanism contributed to the loss in vaccine protection. However, vaccination with Shingrix did not reverse these defects demonstrating that the superior memory durability was not directly related to improved stem-like memory.

Immune protection depends on a diverse TCR repertoire. Both Zostavax and Shingrix vaccinations can recruit new CD4^+^ T cell specificities into the memory compartment, thereby diversifying the repertoire (*33*, *54*). Obviously, this depends on a sizeable naïve compartment, which is largely preserved with aging in CD4^+^ but much less so for CD8^+^ T cells. In addition, the repertoire of memory T cells is under selection pressures, in particular in a setting of latent infection such as VZV. We found VZV gE–specific CD4^+^ T cells to be highly diverse across different subsets, except TEMRA cells, with only a marginal clonality increase in older vaccine recipients. In contrast, VZV gE–specific CD8^+^ T cells were less diverse compared to CD4^+^ T cells already in young adults, consisting with a previous study on the global memory repertoire (*55*). Moreover, they lose diversity with age. Although Shingrix’ adjuvants monophosphoryl lipid A and QS-21 can also stimulate CD8^+^ T cell responses (*32*, *56*), Shingrix vaccination did not restore the age-dependent decline in CD8^+^ T diversity, possibly due to older adults lacking the source of naïve CD8^+^ T cells. Contraction in CD8^+^ T cell diversity may therefore explain the decline in VZV vaccine protection with age, while it does not explain the superior protection by Shingrix.

Instead, Shingrix vaccination prioritizes the generation of T_H_17 CD4^+^ T cells while tending to evade T_reg_ phenotypes. Shingrix likely induces T_H_17 generation through the AS01_B_ adjuvant system, which is also largely responsible for the superior vaccine protection (*57*). AS01_B_ includes the saponin QS-21 and the Toll-like receptor 4 (TLR4) agonist monophosphoryl lipid A. TLR4 activation in CD4^+^ T cells promotes T_H_17 responses in the experimental autoimmune encephalomyelitis (EAE) mouse model (*58*). In addition, TLR4 activation in antigen-presenting cells leads to the production of inflammatory cytokines including IL-6, IL-1β, IL-12, and TNFα, with the potential to skew the milieu for CD4^+^ T helper differentiation from T_reg_- to T_H_17-promoting conditions (*59*, *60*). T_H_17 responses have been associated with effective vaccine responses in other contexts (*61*), such as vaccination with the acellular pertussis vaccine (*16*, *17*, *62*), the adjuvanted VP6 component rotavirus vaccine in mice (*61*), or the T_H_17-skewing, autologous dendritic cell vaccines for patients with ovarian cancer (*63*).

Our data also indicate that T_H_17 heterogeneity and plasticity distinguish durable from short-lived VZV immune memory in older adults. T_H_17 cells that are prone to acquire T_reg_ features appear less protective than those that are poised toward pro-inflammatory T_H_1 phenotypes. This T_H_17 phenotypic spectrum resembles the one previously described for EAE with T_reg_-like phenotypes being consistent with homeostatic T_H_17 cells, while T_H_1-like phenotypes match those of EAE-pathogenic T_H_17 cells (*39*, *45*). In line with a previous report, we find that lipid metabolic signaling is associated with adapting these opposing T_H_17 fates (*41*). More broadly, differentiation of T_H_17 into classical T_reg_ or type 1 regulatory T cells has been described by fate mapping in mice (*40*, *64*, *65*). Unwarranted misdifferentiation of VZV gE–reactive T_H_17 cells into T_reg_ cells may therefore contribute to the higher accumulation of antigen-reactive T_reg_ cells in Zostavax recipients and, thus, to the lower vaccine efficacy and durability of Zostavax. Our observations are supported by our TCR clonotype sharing data and by the fact that virus antigen–specific T_reg_ cells have been described in relatively high proportions in human blood and in multiple contexts such as HIV, HSV (herpes simplex virus), or CMV (*66*) as well as in the skin after VZV challenge (*67*). Thus, the higher T_H_17 numbers combined with their pro-inflammatory, T_H_1-like character in the Shingrix response are associated with superior and durable vaccine protection in older adults.

Our studies have limitations inherent to studying human immunology in a diverse population. The VZV vaccination system is well suited to study the maintenance of immune memory with antigen-specific T cells being protective rather than humoral immunity. We focused on the gE antigen that is shared between the three vaccines, but the immune response is broader for the live-attenuated VZV vaccine (*68*). Because of our direct comparison of rare antigen-specific cells (*29*), we had to rely predominantly on flow cytometry and single-cell sequencing analyses while experimentally testing only selected key results. The interpretation of readout systems used here such as repertoire diversity, differential gene expression, and gene set enrichments should be robust and informative. Our postvaccination time intervals differed in our patient population including between Zostavax and Shingrix, given that Zostavax was discontinued shortly after Shingrix was introduced. It can therefore not completely be excluded that Shingrix recipients eventually develop a similar misdifferentiation phenotype in antigen-specific T_H_17 cells at later time points. Our study populations were small, in part due to the declining number of individuals that had a Zostavax vaccination in recent years. We included male and female participants, but populations were too small to follow up on observations of a sex-specific effect. Still, studying sex differences in the VZV response is particularly relevant, considering recent findings that VZV reactivation increases the risk for dementia, and VZV vaccination can lower this risk particularly in females (*69*–*71*). We found that a type I and II interferon response was defective in VZV-reactive CD4^+^ T cells of older adults, which could be restored by Shingrix vaccination only in female vaccine recipients. We also noted trending differences in subset heterogeneity between male and female vaccinees. Last, given the substantial heterogeneity of major histocompatibility complex polymorphism in human populations, we had to rely on a functional definition of antigen specificity on the basis of the expression of activation markers. Our approach relied on peptide stimulation ex vivo, and it is possible that because of the cytokine milieu in these cultures (*72*), CD8^+^ T cells become bystander activated and are copurified in our experiments. Nonetheless, such a contamination would not explain the differences that we observed by comparing different study groups. Our study indicates the potential of leveraging T_H_17 CD4^+^ memory T cell responses in vaccination strategies for older adults. Inclusion of T_H_17-inducing adjuvants, such as AS01_B_, in vaccines would be a relevant area for future research and development.

## MATERIALS AND METHODS

### Study design and study populations

In this study, we sought to determine the molecular determinants of T cell memory durability in older adults and used VZV vaccination as a model system. We contrasted VZV gE–specific T cells derived from peripheral blood of (i) young adults (<30 years) who received a childhood live-attenuated VZV vaccine, Varivax, which elicits long-lasting immune memory and protection against VZV; (ii) older adults (>55 years) who received the same live-attenuated vaccine strain, marketed as Zostavax, which induces a short-term, partial protection against VZV reactivation; and (iii) older adults (>55 years) who received an effective, durable adjuvanted vaccine, Shingrix.

We recruited a total of 53 volunteers who did not have an acute or active chronic disease, cancer, or autoimmune disease; who were not on a chronic anti-inflammatory medication; and for whom the vaccination history was documented. Recruitment was done by the Mayo Clinic Biobank registry. The sample size of Zostavax recipients was restricted by the number of individuals in the registry who fulfilled the inclusion criteria and agreed to participate. A similar number of Shingrix recipients were chosen who had received the vaccine early after it became available. We received the peripheral blood of these volunteers and isolated PBMCs and serum. All participants gave informed written consent. Chronic diseases were permitted if controlled by medication. Phlebotomy was scheduled in the morning, and volunteers were asked to fast before the blood collection. Volunteers for single-cell sequencing were recruited in 10 batches between December 2021 and July 2023. Subject information on samples used in single-cell sequencing experiments is summarized in tables S1 and S2. In addition, PBMCs from leukoreduction system chambers of 37 blood donors were purchased from the Mayo Clinic Blood Donor Center, Department of Laboratory Medicine and Pathology–Component Laboratory. Samples were deidentified, except for age and self-assigned sex information. These samples were only used for mechanistic studies. Samples from male and female individuals were used. Young adults were 20 to 35 years old, and older adults were 60 years or older. Studies involving human subjects were approved by the Mayo Clinic Institutional Review Board (study number no. 21-005196).

### Cell isolation, culture, and antigen stimulation

Lymphoprep (Stemcell Technologies, no. 07861) was used to purify PBMCs by density centrifugation according to the manufacturer’s instructions. Cells were used fresh in all experiments and cultured in RPMI 1640 medium supplemented with 5% filtered human AB serum (Sigma-Aldrich, no. H4522), penicillin (100 U/ml), and streptomycin (100 U/ml; Sigma-Aldrich, no. P0781) in an incubator with 5% CO_2_ and atmospheric O_2_. For peptide stimulation experiments, we performed AIM assays (*30*). Briefly, PBMCs were seeded at 1 × 10^6^ cells per U-bottom 96-well plate (for flow cytometry analyses) or 20 × 10^6^ per 12-well plate (for single-cell sequencing) and stimulated with a VZV gE peptide pool (1 μg/ml) diluted in 0.2% DMSO (15-mer peptides with 11–amino acid overlap, total of 153 peptides; no. PM-VZV-gE, JPT Peptide Technologies). The ultra-LEAF anti-CD28 antibody was added at 1 μg/ml (clone 28.2, BioLegend, no. 302943). For samples that were subjected to single-cell sequencing or flow cytometry to measure CD40L levels, an anti-CD40 blocking antibody was added at 1 μg/ml (clone HB14, Miltenyi, no. 130-094-133). Parallel control PBMC cultures of the same individuals received 0.2% DMSO instead of the VZV gE peptide mix or SEB (1 μg/ml; Toxin Technologies, no. BT202red). PBMCs were spun in plates at 300*g* for 3 min before culturing for 42 hours. To calculate net frequences, the proportion of activated T cells in the solvent control cultures was subtracted from the proportion of antigen-responsive T cells. Quantitation and phenotyping of VZV gE–reactive T cells after 42 hours of VZV gE stimulation were performed via cell surface antibody staining or intracellular staining. The used antibodies are listed in table S4. Viability dye Live/Dead Fixable Blue (Invitrogen, no. L23105) was included. For intracellular staining, cells were fixed with the eBioscience Foxp3/Transcription Factor Staining Buffer Set (Thermo Fisher Scientific, no. 00-5523-00) according to the manufacturer’s instructions. Most experiments were analyzed on a 5-laser Cytek Aurora (Cytek Biosciences) running SpectroFlo v3 with UltraComp eBeads compensation beads (Thermo Fisher Scientific, no. 01-2222-42). Only optimization experiments for the AIM assays were analyzed on a BD LSRFortessa X-20.

### CMV serostatus assessment

Serum was isolated from whole blood, which was collected in serum separator tubes. Blood was centrifuged at 800*g* for 10 min, and the serum supernatant was collected. Serum was aliquoted and frozen at −80°C until use. To test for CMV serostatus, we used the CMV IgG EIA Kit (Bio-Rad, no. 25177) according to the manufacturer’s instructions.

### Cell preparation for single-cell sequencing

For single-cell sequencing experiments, 42 hours after peptide stimulation, cultures were prepurified with the EasySep Human T Cell Isolation Kit (Stemcell Technologies, no. 17951). T cells were counted and resuspended at 1 × 10^6^ to 2 × 10^6^ cells/50 μl and blocked with TruStain FcX Fc Blocking reagent (BioLegend, no. 422301) for 10 min at 4°C before incubation with hashtag TotalSeq-B antibodies (0.5 μl per 100 μl of cell suspension; table S5) and flow cytometry antibodies (table S4; Live/Dead Fixable Aqua, Invitrogen, no. L34957) for 30 min at 4°C. Cells were washed once before FACS on a BD FACSAria 4-laser digital flow cytometer with FACSDiva v8 software. Cell sorting was performed by the Mayo Clinic Microscopy and Cell Analysis Core Flow Cytometry Lab. Live, single CD3^+^ CD8^+^ or CD3^+^ CD4^+^ cells that were CD69^hi^ and/or CD137^+^ were collected. A representative gating strategy is shown in fig. S12. After sorting, cells were counted and pooled into one sample across T cell subsets and donors at similar cell numbers: VZV gE–responsive CD4^+^ T cells, DMSO background–activated CD4^+^ T cells, VZV gE–responsive CD8^+^ T cells (at about half the proportion of VZV gE–responsive CD4^+^ T cells), and DMSO background–activated CD8^+^ T cells. The pooled sample was then stained with diluted TotalSeq-B cell surface marker antibodies (BioLegend; table S5) in phosphate-buffered saline (PBS) containing 2% fetal bovine serum for 30 min at 4°C. Cells were washed twice with PBS containing 2% fetal bovine serum, and cell viability was determined with Trypan Blue, which was consistently >90%. Cells were subjected to single-cell capture via the Chromium controller (10x Genomics), Chromium Next GEM Single Cell 3′ Kit v3.1 (Single Index, 10x Genomics, no. PN-1000121) and gel beads (10x Genomics, no. 1000376). About 20,000 cells per pooled samples were targeted for cell capture. Three libraries were generated from each captured sample: 3′ gene expression library, library of antibody-derived tags (ADTs) for TotalSeq-B antibodies including hashtag antibodies and cell surface marker (3’ Feature Barcode Library Kit, 10x Genomics, no. PN-1000079) collectively known as CITE-seq (*73*). The third library was constructed to capture TCR CDR3 sequences via a custom protocol (see the section below).

### Functional testing of VZV gE–reactive T cell subsets

PBMCs were stimulated with VZV gE peptides for 42 hours as above. CD4^+^ T cells were purified from these cultures via the EasySep Human CD4^+^ T Cell Isolation Kit (Stemcell Technologies, no. 17952). Cells were stained with flow cytometry antibodies, and VZV gE–reactive T cell subsets were FACS collected via a BD FACSAria. Cells were pregated to be live, single CD3^+^ CD4^+^ cells that were CD69^hi^ and/or CD137^+^ and further subdivided via single-cell cluster–defining markers: CCR6^+^ CCR4^−^ IL-7R^+^ (cluster 0/T_H_1), CCR6^+^ CCR4^+^ TIGIT^+^ (cluster 1/T_reg_), and CCR6^+^ CCR4^+^ CD26^+^ TIGIT^−^ (cluster 4/T_H_17). A representative gating strategy is shown in fig. S13. Sorted cells were stained with 2 μM CellTrace Violet (Invitrogen, no. C34557) according to the manufacturer’s instructions. Cells were counted and seeded into 96-well plates (100,000 cells per well, 100-μl volume) that had been coated with αCD3/αCD28 antibodies (both 1 μg/ml; clones UCHT1 and CD28.8, respectively; BioLegend, nos. 300465 and 302943). After 5 days of culture, the culture supernatant was collected and frozen at −80°C. Cytokine levels in these culture supernatants were measured with the LEGENDPlex 12-plex Human Th Cytokine Panel (BioLegend, no. 741028) according to the manufacturer’s instructions. Cells were also collected and stained with flow cytometry antibodies (table S4), viability dye Live/Dead Fixable Blue (Invitrogen, no. L23105), and ApoTracker Green (BioLegend, no. 4274020). Samples were analyzed on a 5-laser Cytek Aurora (Cytek Biosciences). UltraComp eBeads compensation beads (Thermo Fisher Scientific, no. 01-2222-42) were used for single staining controls.

### Single-cell sequencing and data processing

Single-cell gene expression and ADT libraries were sequenced on a NovaSeq 6000 S4 (Illumina) and 150-cycle, paired-end kit to target depths of 50,000 read pairs per cell for gene expression and 5000 read pairs per cell for feature barcodes of the ADT/CITE libraries. Cell capture and library construction were performed by the Mayo Clinic Medical Genome Facility Genome Analysis Core, and sequencing was performed by Novogene Corporation or Azenta Life Sciences. Data were similarly processed as described previously. Fastq reads were assessed using FastQC version 0.11.9. Following quality assessment, raw reads were aligned to the GRCh38 human genome using Cell Ranger multi version 7.1.0. The Cell Ranger multi pipeline was used to quantify libraries of ADTs and to demultiplex hashtagged samples of individual donors in pooled libraries. Seurat version 4.2.1 was used for downstream analysis following data preprocessing (*74*, *75*). Quality control was conducted to exclude low-quality cells on the basis of the following filters: cells with <200 or >5000 expressed genes, >10% unique molecular identifier (UMI) mapping to mitochondrion-encoded transcripts, or >20,000 ADT UMIs. DoubletFinder was applied to identify and remove cell doublets. We identified CD4^+^ versus CD8^+^ T cells on the basis of their CD4 and CD8α ADT protein as well as *CD4* and *CD8A* gene expression. CD4^+^ T cells were CD4 ADT–positive but CD8α ADT–negative or *CD4* RNA–expressing but *CD8A* RNA–negative. CD8^+^ T cells were CD8α ADT–positive but CD4 ADT–negative or *CD8A* RNA–expressing but *CD4* RNA–negative. Across all experiments and groups, we obtained 137,461 cells, of which 97,663 were CD4^+^ T cells and 39,798 were CD8^+^ T cells. CD4^+^ and CD8^+^ T cells were further processed separately. For gene expression normalization, individual samples were processed using SCTransform while regressing out mitochondrial gene expression. ADT counts were normalized using centralized log-ratio transformation. Batches across experiments were integrated using diagonalized canonical correlation analysis to identify mutual nearest neighbors that act as anchors using the FindIntegrationAnchors and IntegrateData functions (*75*). The integrated gene expression and ADT modalities and respective matrices were scaled, and principal components were calculated. A weighted nearest-neighbor graph was constructed using the FindMultiModalNeighbors function (*74*) and used to generate the UMAP embeddings, facilitating the visualization for both modalities. Clustering was performed on the basis of the weighted nearest neighbor using the smart local moving algorithm (*74*). We performed imputation of gene expression using MAGIC (Markov Affinity-based Graph Imputation of Cells) solely for data visualization. To assist in annotations of clusters to classical T cell subsets, we performed UCell gene module scores using the AddModuleScore_UCell function with published T cell gene signatures (*76*, *77*). Pseudotime trajectory analysis was performed with SlingShot version 2.14.0 using CD4^+^ T cell cluster C4 as the starting point.

### Pseudobulk differential expression analysis

Raw counts of samples were aggregated by summing the UMIs from their respective single cells using Seurat’s built-in function AggregateExpression (*74*). Samples containing fewer than 20 cells were excluded from this analysis. Low-expressing genes with fewer than 10 counts in the selected comparisons were removed using the filterByExpr function (*78*). The remaining raw counts were transformed using regularized log transformation to compute principal components, followed by batch effect correction with plotPCA and removeBatchEffect functions, respectively (*78*, *79*). DEGs were identified by comparing age and vaccine groups across all cells, within a given cluster as well as between the clusters. Only clusters with minimum sample representation of two male and two female participants per group were included. DEGs were identified by modeling the mean-variance trend on log-transformed counts per million of each gene using voomWithQualityWeights from the limma package. For Shingrix and Zostavax comparisons, the experimental batch was incorporated as a covariate. Contrasts were fitted to the model, and empirical Bayes moderation was applied to identify DEGs with an adjusted *P* value <0.05 (tables S6 and S7). Pathway enrichment analyses on DEGs were conducted with EnrichR focusing on Gene Ontology, Reactome, BioPlanet, Kyoto Encyclopedia of Genes and Genomes, MSigDB (Molecular Signatures Database), and WikiPathways gene sets. GSEA was performed by FGSEA version 1.20.0 for adaptiveness and lymphocyte innateness genes (*31*), exhaustion-associated genes (GSE9650 and GSE41867), cellular senescence–associated genes [Reactome R-HSA-2559583, SenMayo (*80*)], T helper signatures (*76*, *77*), and SREBP-related genes (Reactome R-HSA-2426168, WikiPathways WP1982). Enrichment analyses were performed with custom background gene lists, which included all expressed genes in each cell type. Gene sets can also be found in table S8.

### Single-cell TCR sequencing and data analysis

Single-cell libraries for TCR sequences were created from the same 3′ single-cell, full-length cDNA material used for gene expression and ADT libraries. *TRB* libraries were generated as described previously (*81*–*83*). For one experiment, we also generated *TRA* libraries. Libraries were sequenced on MiSeq or NextSeq 2000 sequencers (Illumina) with a 100-cycle single-end kit with a TruSeq Read 1 primer and custom index 1 primer (cycle 28-100-0-0). We targeted at least 2500 read pairs per cell. Fastq files were processed with WAT3R (*84*) to identify the TCR chains and CDR3 sequences. We obtained a *TRB* CDR3 sequence from 38% of CD4^+^ or CD8^+^ T cells that were included in our transcriptomic analyses. We calculated the Inverse Simpson index to estimate TCRβ diversity and the Gini index to calculate TCRβ clonality, as described previously (*29*). For clonotype tracking across T cell subsets, we calculated the TCR repertoire similarity score (TRSS) (*40*). Only samples with at least 10 expanded clones in a given sample were considered.

### RNA isolation and quantitative reverse transcription polymerase chain reaction

T cells were washed with PBS, the cell pellet was lysed in RLT buffer, and RNA was isolated with the RNeasy Micro kit (Qiagen, no. 74004) according to the manufacturer’s instructions but avoiding the deoxyribonuclease digestion step. About 200 ng of RNA was used for reverse transcription via the SuperScript VILO cDNA Synthesis Kit (Invitrogen, no. 11754050). The PowerUp SYBR Green Master Mix (Applied Biosystems, no. A25742) was used for qPCR analyses on a QuantStudio 6 (Applied Biosystems). Primers were as follows: *FOXP3*: forward, 5′-GAAACAGCACATTCCCAGAGTTC-3′; *FOXP3*: reverse, 5′-ATGGCCCAGCGGATGAG-3′; *PDCD1*: forward, 5′-AAGGCGCAGATCAAAGAGAGCC-3′; *PDCD1*: reverse, 5′-CAACCACCAGGGTTTGGAACTG-3′; *RBPJ*: forward, 5′-TCATGCCAGTTCACAGCAGTGG-3′; *RBPJ*: reverse, 5′-TGGATGTAGCCATCTCGGACTG-3′; *TOX*: forward, 5′-CGCTACCTTTGGCGAAGTCTCT-3′; *TOX*: reverse, 5′-CTGGCTCTGTATGCTGCGAGTT-3′. *ACTB* was used as the reference gene: forward, 5′-CACCATTGGCAATGAGCGGTTC-3′; reverse, 5′-AGGTCTTTGCGGATGTCCACGT-3′.

### Statistical analyses

For the statistical association of cluster proportions with vaccine groups, we performed probability vector analyses and permutation tests, as described previously (*29*). Briefly, each sample is characterized as a probability vector whose components are the relative cell frequency in each of the clusters and the cardinality represents the number of distinct clusters. We performed linear regression analyses with sex adjustment between the individual components of the probability vector to examine the association between the probability vector and vaccine groups. The association with the entire probability vector is summarized by the sum of squares of all *z*-scores corresponding to the regression coefficient of the vaccine groups from the linear regression analyses and tested via a permutation test permuting the vaccine groups.

All other statistical analyses were performed via Prism software 10 (GraphPad). Data are presented as the means with error bars indicating the standard error of the mean (SEM), unless otherwise indicated. Unpaired or paired, two-tailed Student’s *t* tests were used when comparing two groups. A one-way or two-way analysis of variance (ANOVA) with multiple comparisons test was performed for multigroup comparisons. A *P* value less than 0.05 was considered statistically significant. Significance levels (*P < 0.05, **P < 0.01, ***P < 0.001, and ****P < 0.0001) are indicated in figures.
