# Supplementary material for: Antigen-specific TH17 cells offset the age-related decline in durable T cell immunity
Source: Sci Adv. 2026 Feb 6;12(6):eaea7131. doi: 10.1126/sciadv.aea7131 (PMC12880537; doi:10.1126/sciadv.aea7131)
Supplement: Supplementary file 1 — Figs. S1 to S13 Tables S1 and S2 Legends for tables S3 to S8 [file sciadv.aea7131_sm.pdf]

Science Advances
AAAS

Supplementary Materials for

Antigen-specific T_H17 cells offset the age-related decline in durable T cell immunity

Ines Sturmlechner *et al.*

Corresponding author: Jörg J. Goronzy, goronzy.jorg@mayo.edu; Ines Sturmlechner, sturmlechner.ines@mayo.edu

Sci. Adv. **12**, eaea7131 (2026)
DOI: 10.1126/sciadv.aea7131

The PDF file includes:

Figs. S1 to S13
Tables S1 and S2
Legends for tables S3 to S8

Other Supplementary Material for this manuscript includes the following:

Tables S3 to S8

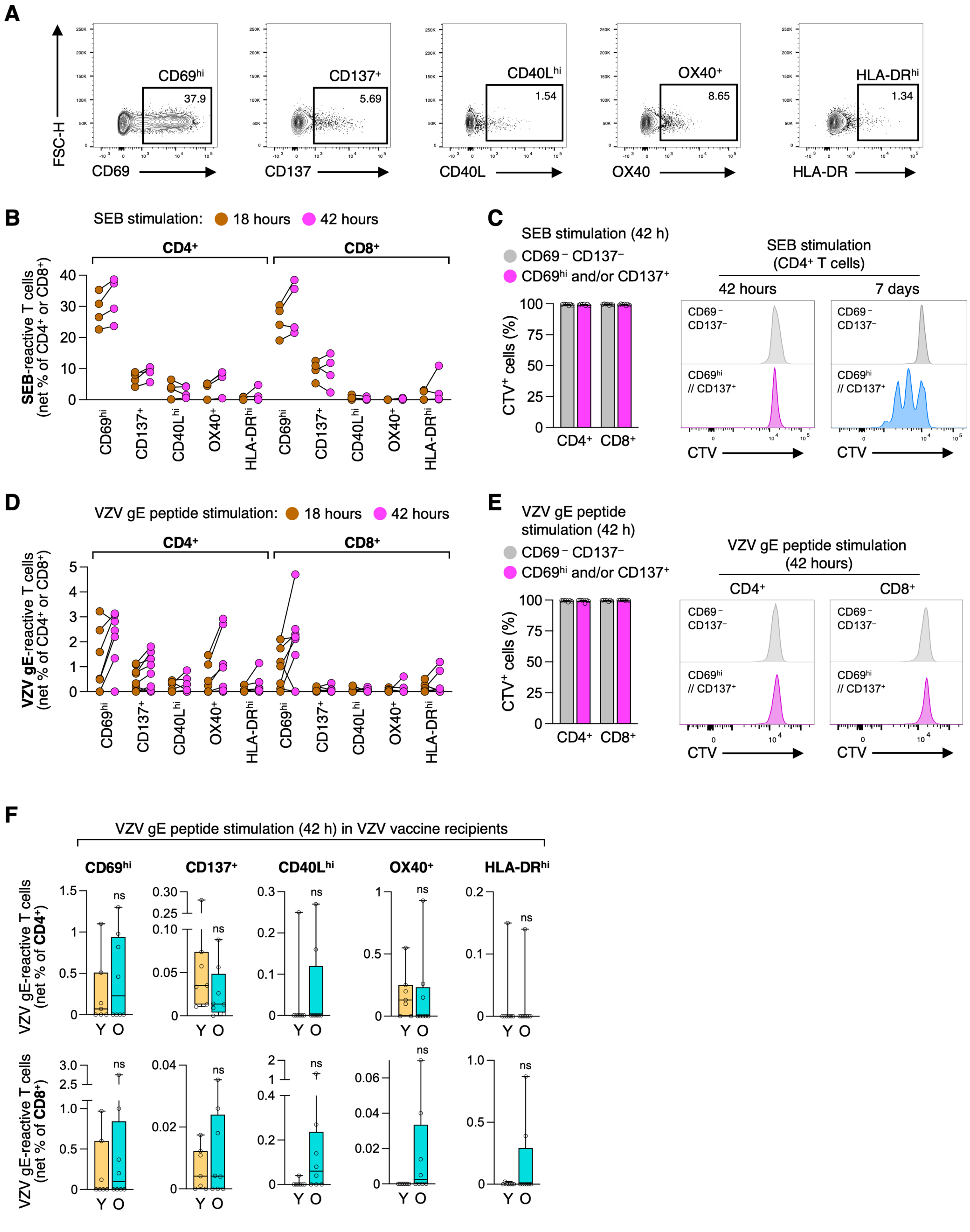

Figure S1, related to Fig. 1: Young and older vaccine recipients have similar frequencies of VZV gE-reactive T cells. The activation-induced marker assay was optimized with the goal to

comprehensively identify the maximum number of VZV gE-responsive T cells in human peripheral blood. (**A**) Representative flow cytometry gating of activation markers after SEB (Staphylococcal Enterotoxin B) stimulation for 42 hours. (**B**) SEB-reactive $CD4^+$ and $CD8^+$ T cells in human peripheral blood identified by indicated activation markers at 18 or 42 hours after stimulation. Data show background control-subtracted (net) frequencies. (**C**) CellTrace dilution assays to confirm the absence of T cell division at 42 hours after SEB stimulation. A 7-day timepoint was added as positive control. (**D**) AIM assay of VZV gE-reactive $CD4^+$ and $CD8^+$ T cells. (**E**) CellTrace dilution assays at 42 hours after VZV gE stimulation. (**F**) Frequencies of VZV gE-reactive $CD4^+$ and $CD8^+$ T cells in young (Y) and older (O) VZV vaccine recipients as identified by AIM assays. Data show background control-subtracted (net) frequencies. Data show mean ± SEM (C and E) or median (F). All datapoints represent distinct biological replicates, and data were pooled from 2 (B) or 3 (C to D) independent experiments Data were compared by Mann-Whitney tests (F). ns, not significant.

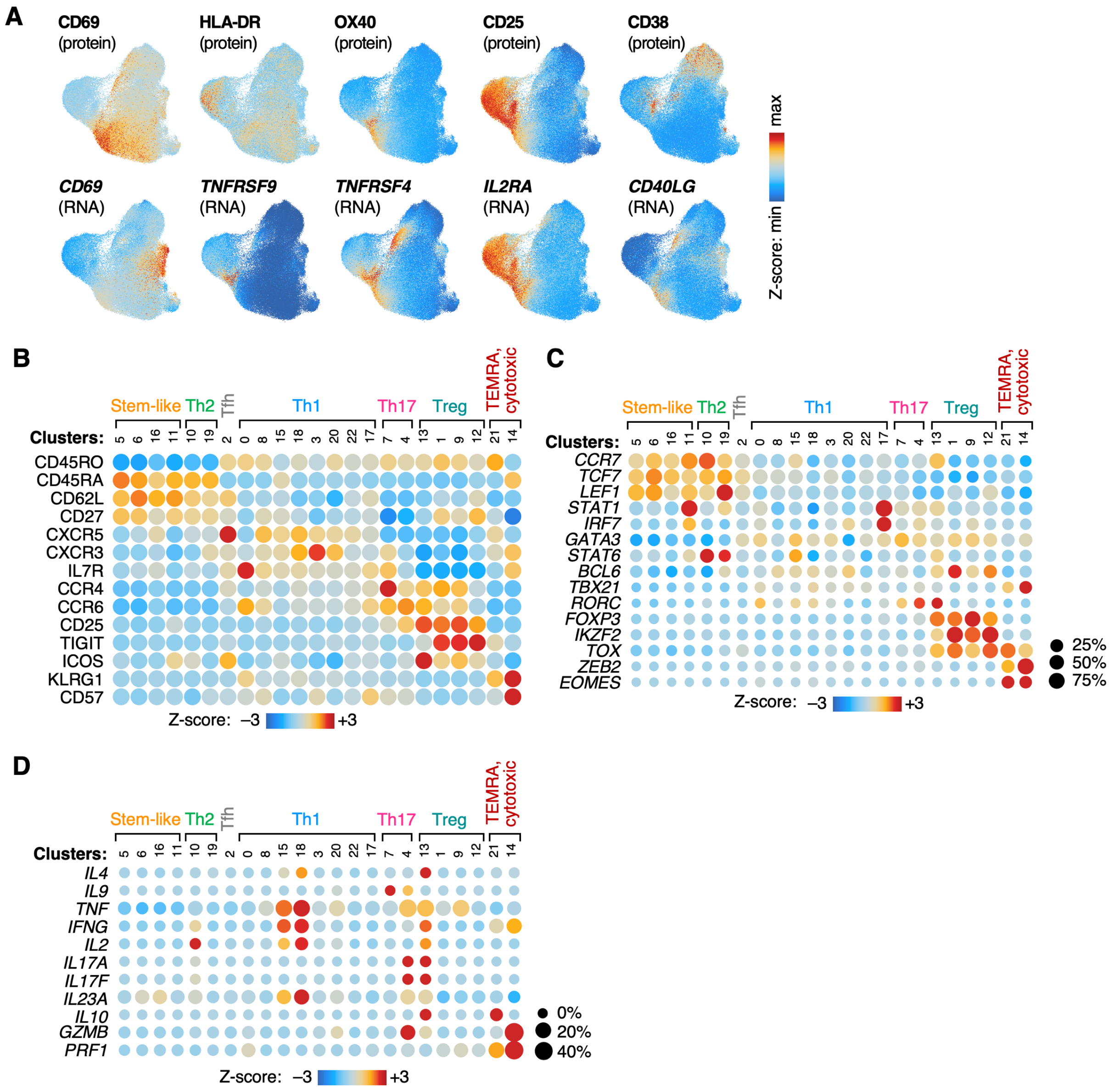

Figure S2, related to Fig. 1: Cluster and subset definition of VZV gE-reactive CD4$^+$ T cells. (**A**) Feature plots of activation markers based on CITE protein or gene expression levels. (**B**) Dotplot heatmap of CITE protein levels across VZV gE-reactive CD4$^+$ T cell clusters showing classical T cell subset markers. (**C**) as in (B) but for gene expression levels of *CCR7* and transcription factors. The dot size corresponds to the proportion of cells expressing the indicated gene. (**D**) as in (C) but for gene expression levels of cytokines and effector molecules.

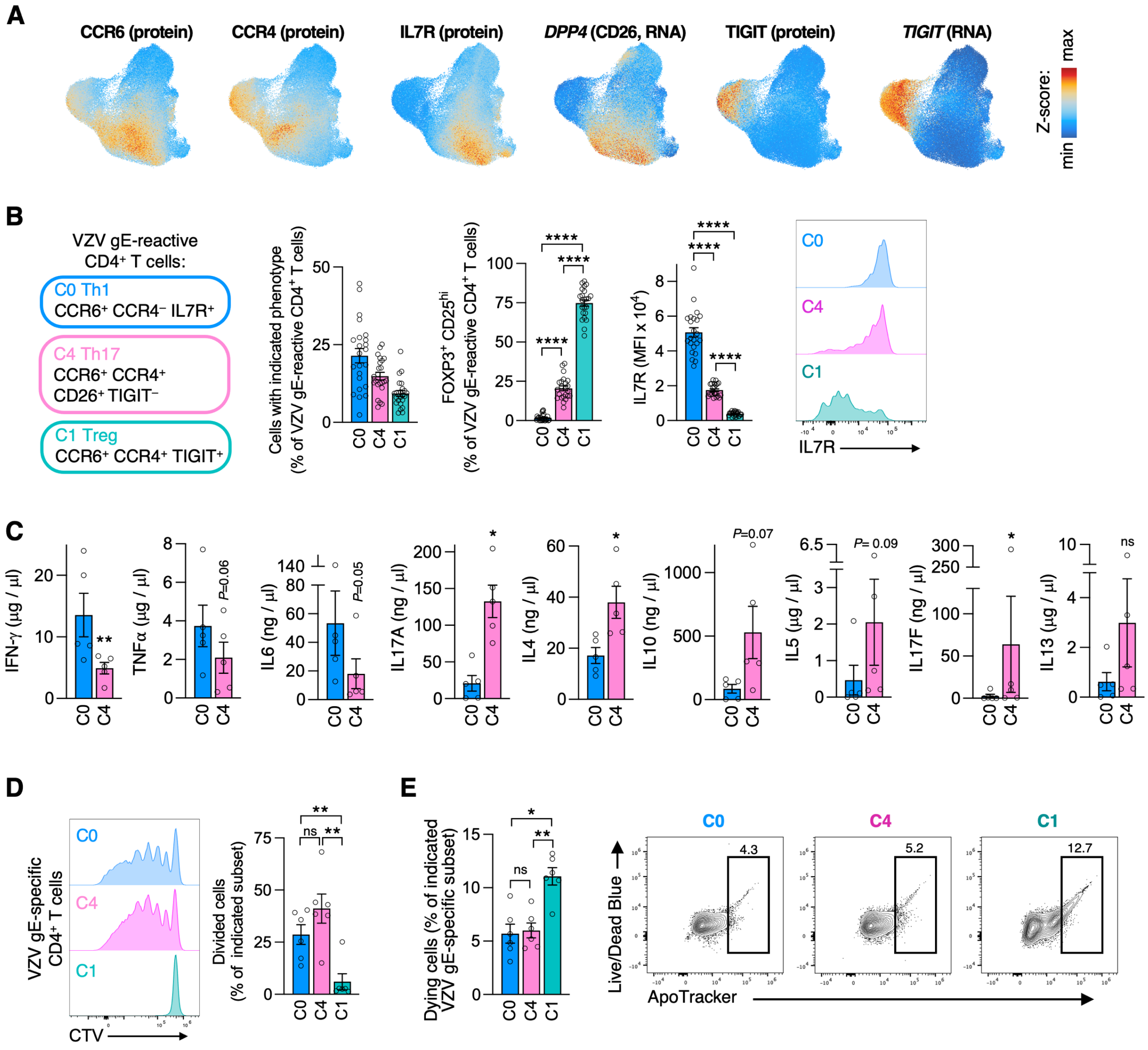

Figure S3, related to Fig. 1: CD4$^+$ memory T cells specific for VZV gE are phenotypically highly diverse. (**A**) Feature plots of cluster markers used for FACS experiments. (**B**) Flow cytometry of VZV gE-stimulated T cells, examining the expression of FOXP3, CD25, and IL7R in subsets corresponding to single cell sequencing clusters C0, C1, C4. (**C**) PBMCs of older adults were stimulated with VZV gE peptides. Activated CD4$^+$ T cells with a Th1 (corresponding to C0), Th17 (corresponding to C4), and Treg phenotype (corresponding to C1) were collected. Sorted cells were re-stimulated with αCD3/αCD28 antibodies for 5 days. Cytokine production was measured by LegendPlex multiplex cytokine assays. (**D**) VZV gE-reactive CD4$^+$ T cell subsets

were collected as in (C) and stained with CellTrace Violet (CTV) before a 5-day αCD3/αCD28 antibody stimulation. (**E**) Proportion and representative flow cytometry plots of dying cells marked by ApoTracker and viability dye Live/Dead Blue in cultures as in (D). Data show mean ± SEM (B to E). All datapoints represent distinct biological replicates and data from 4 independent experiments are pooled (C to E). Data were compared by one-way ANOVA with Tukey's multiple comparisons (B, D, and E), two-tailed, paired *t*-test (C). *P<0.05, **P<0.01, ***P<0.001, ****P<0.0001. ns, not significant.

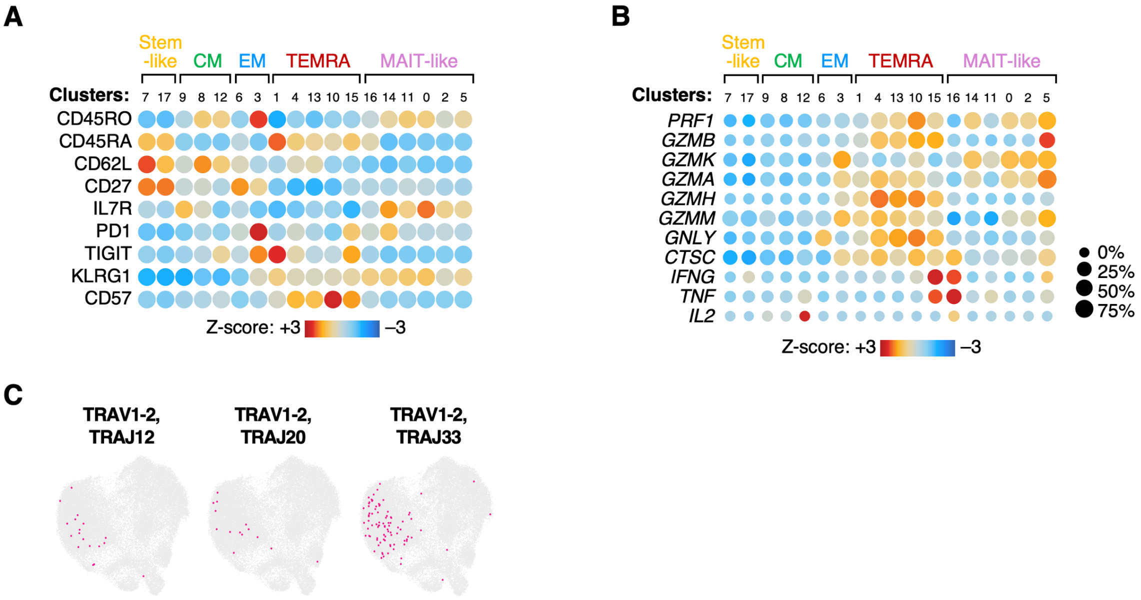

Figure S4, related to Fig. 1: The VZV gE-reactive CD8$^+$ memory T cell response includes diverse subsets. (**A**) Dotplot heatmap of CITE protein levels across VZV gE-reactive CD8$^+$ T cell clusters showing classical T cell subset markers. (**B**) Dotplot transcriptomic heatmap of effector molecules across clusters. The dot size corresponds to the proportion of cells expressing the indicated gene. (**C**) CD8$^+$ T cells expressing indicated TRAV-TRAJ segments (pink colored) typically found in MAIT cells.

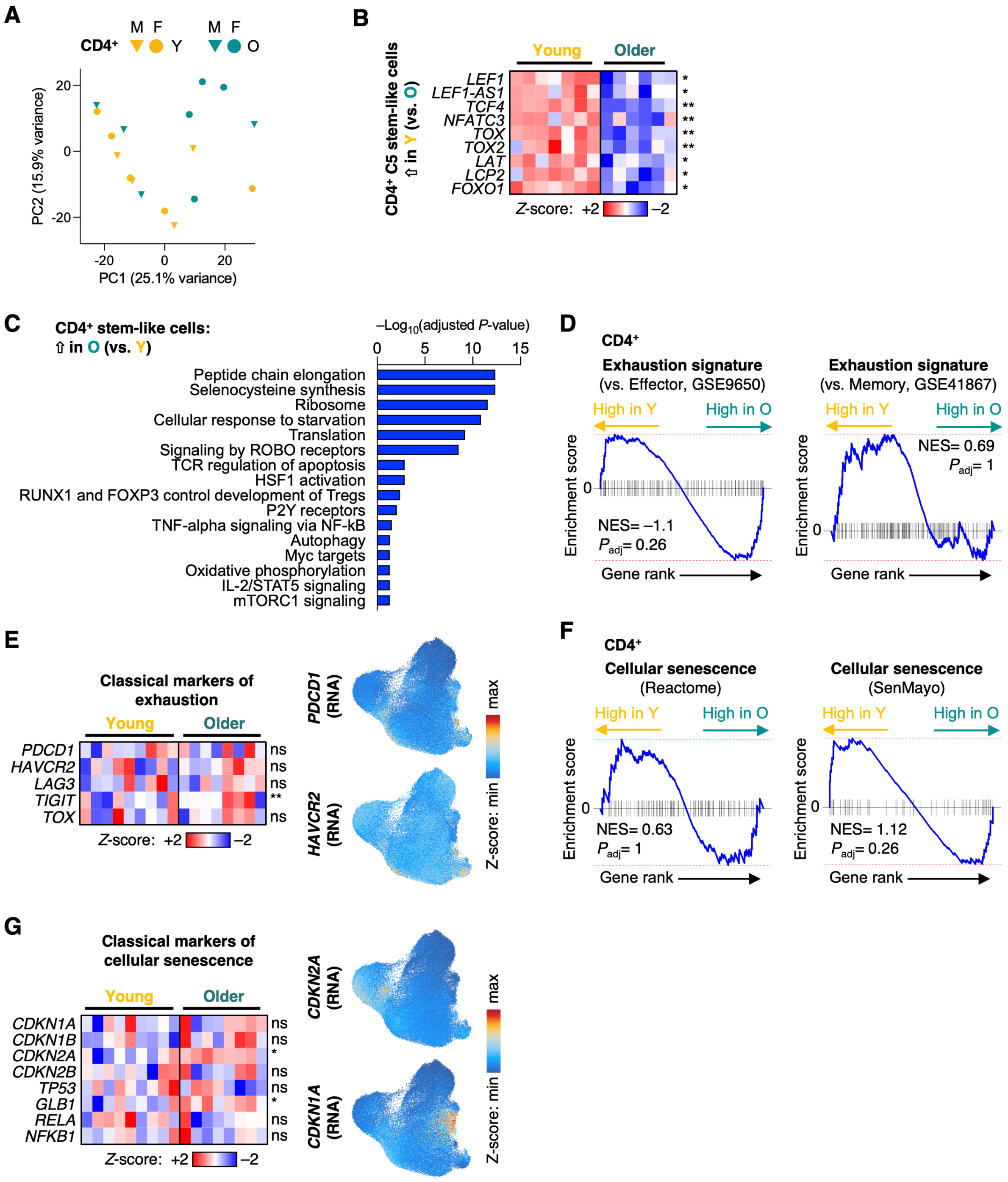

Figure S5, related to Fig. 3: Stem-like features in the CD4$^+$ T cell memory response from older adults against VZV gE are diminished. (**A**) Principal component analysis of CD4$^+$ VZV gE-specific single cell transcripts aggregated into pseudo-bulk data for each vaccine recipient (M, male, and F, female). (**B**) Heatmap of pseudo-bulk gene expression of selected CD4$^+$ T cell DEGs

in C5 stem-like cells. Only clusters with a minimum sample representation of 2 male and 2 female participants per group were included. (**C**) Pathway enrichment of $CD4^+$ C5 DEGs which were higher expressed in O than in Y. (**D**) GSEA on gene expression data in O versus Y for T cell exhaustion gene sets. (**E**) Heatmap of pseudo-bulk gene expression (left) and feature plots (right) of classical markers of T cell exhaustion. (**F**) GSEA as in (D) for cellular senescence gene sets. (**G**) Heatmap and feature plots of classical cellular senescence markers.

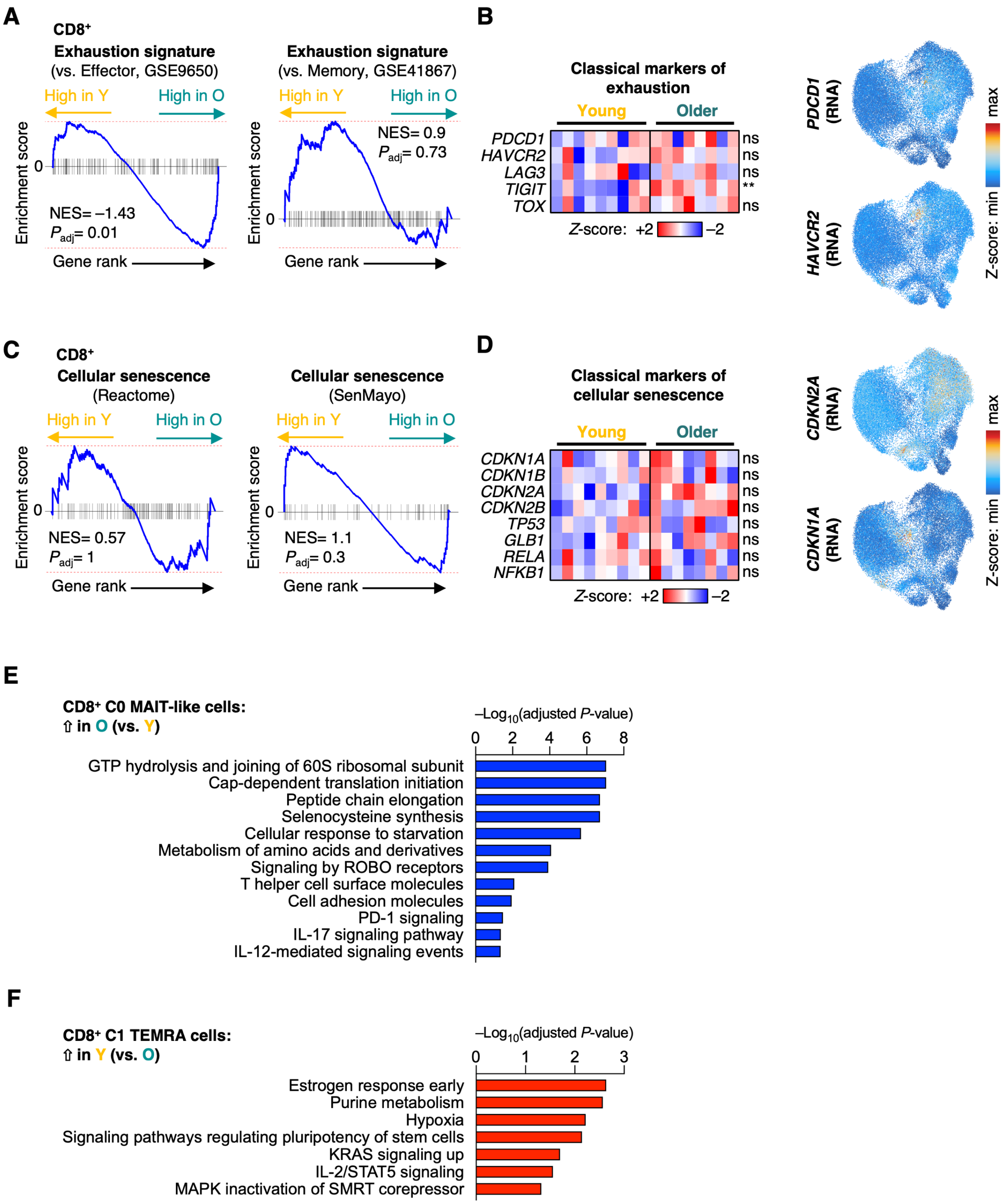

Figure S6, related to Fig. 3: CD8$^+$ memory T cells specific to VZV gE lack signatures of age-related exhaustion or cellular senescence. (**A**) GSEA of T cell exhaustion gene sets on pseudo-bulk gene expression data of total VZV gE-reactive CD8$^+$ T cells in Y versus O vaccine recipients. (**B**) Heatmap of pseudo-bulk gene expression (left) and feature plots (right) of classical

markers of T cell exhaustion. (**C**) GSEA of cellular senescence gene sets as in (A). (**D**) Heatmap of pseudo-bulk gene expression (left) and feature plots (right) of classical cellular senescence markers. **(E)** Pathway enrichment for CD8^{+} C0 DEGs which were higher expressed in O than in Y. (**F**) Pathway enrichment for CD8^{+} C1 DEGs which were higher expressed in Y than in O.

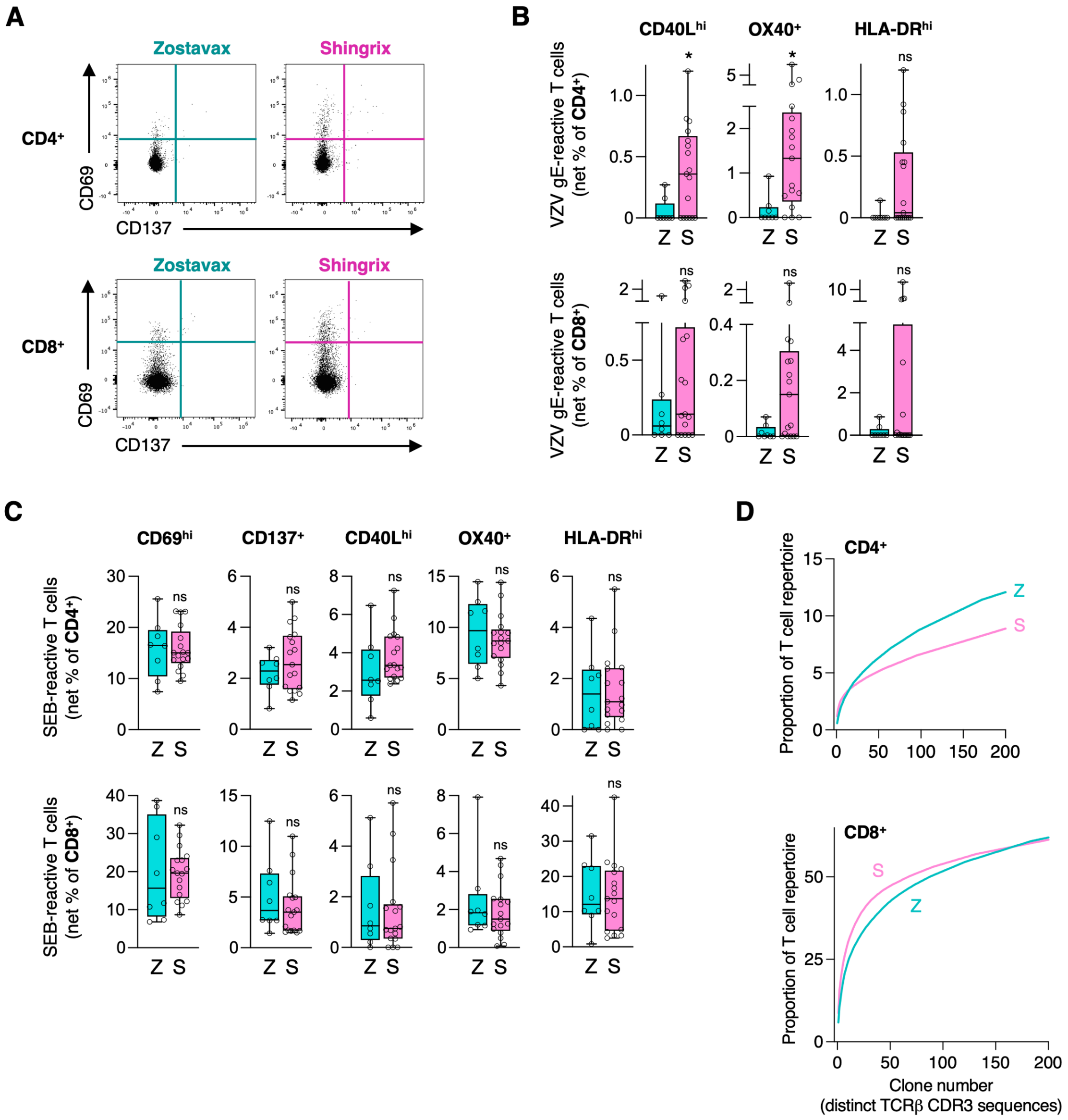

Figure S7, related to Fig. 4: VZV gE responses are increased in Shingrix recipients but are not more diverse. (**A**) Representative flow cytometry plots of CD69^{hi} and/or CD137^{+} T cells after VZV gE peptide stimulation. (**B**) Proportion of VZV gE-reactive CD4^{+} or CD8^{+} T cells in Zostavax (Z) or Shingrix (S) vaccine recipients as identified by flow cytometry for activation markers CD40L^{hi}, OX40^{hi} or HLA-DR^{hi}. Data show background control-subtracted (net) frequencies. Data from 8 independent experiments were pooled. (**C**) Proportion of SEB-reactive CD4^{+} or CD8^{+} T cells in Z or S vaccine recipients as identified by indicated activation marker in flow cytometry analysis. Data show background control-subtracted (net) frequencies. Data from 8 independent

experiments were pooled. (**D**) Cumulative TCR frequency plots for VZV gE-specific $CD4^+$ (top) and $CD8^+$ T cells (bottom) from Z and S recipients in single cell sequencing experiments. The plots show TCRs ranked by their descending clone sizes versus the space they occupy. Data show median (B and C). All datapoints represent distinct biological replicates. Data were compared by Mann-Whitney tests (B and C). *P<0.05. ns, not significant.

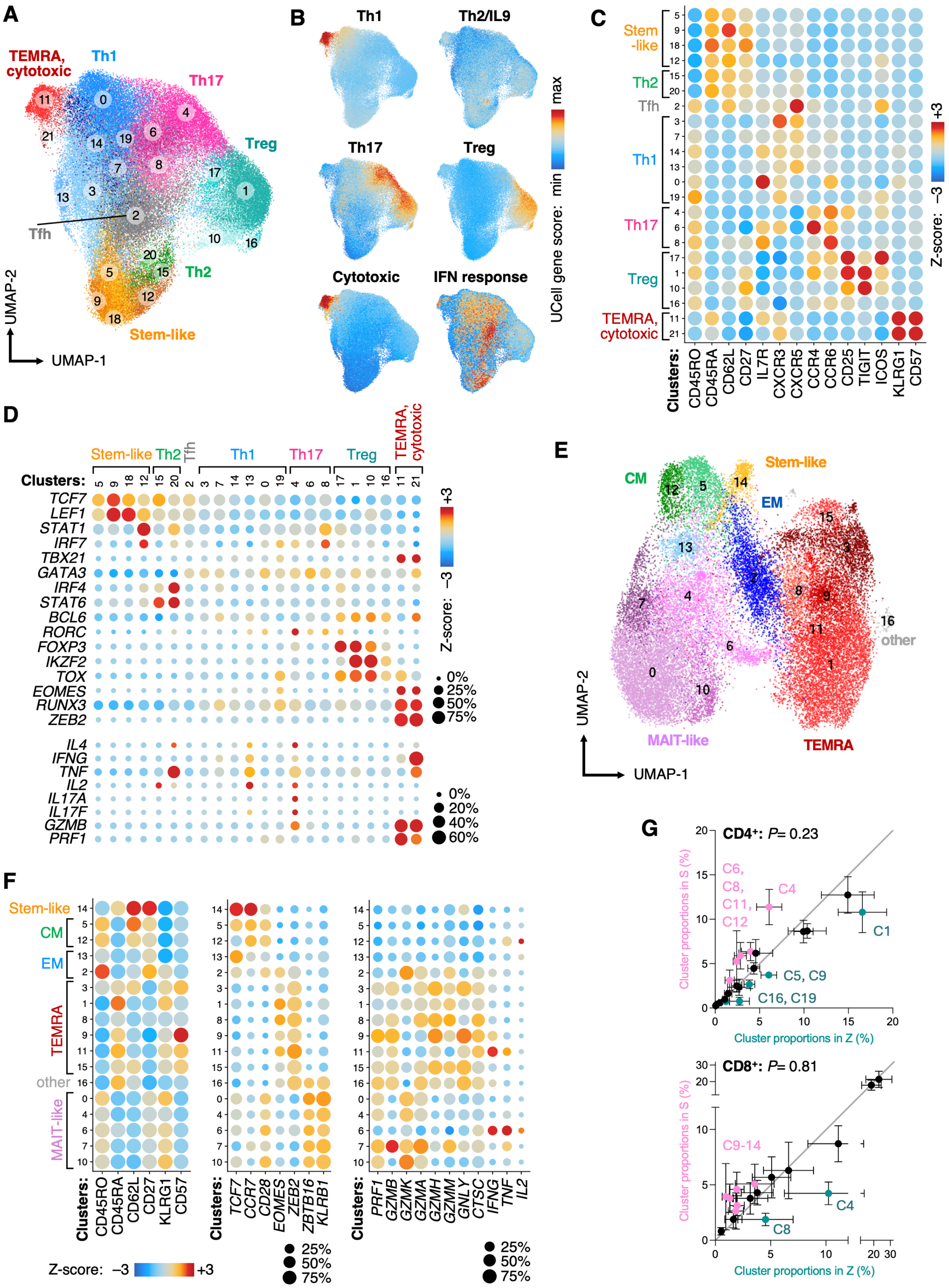
A
TEMRA, cytotoxic
Th1
Th17
Treg
Tfh
Th2
Stem-like
UMAP-2
UMAP-1
B
Th1
Th2/IL9
Th17
Treg
Cytotoxic
IFN response
UCell gene score: min max
C
Stem -like
Th2
Tfh
Th1
Th17
Treg
TEMRA, cytotoxic
Clusters:
CD45RO
CD45RA
CD62L
CD27
IL7R
CXCR3
CXCR5
CCR4
CCR6
CD25
TIGIT
ICOS
KLRG1
CD57
Z-score: −3 +3
D
Stem-like Th2 Tfh Th1 Th17 Treg TEMRA, cytotoxic
Clusters:
TCF7
LEF1
STAT1
IRF7
TBX21
GATA3
IRF4
STAT6
BCL6
RORC
FOXP3
IKZF2
TOX
EOMES
RUNX3
ZEB2
IL4
IFNG
TNF
IL2
IL17A
IL17F
GZMB
PRF1
Z-score: −3 +3
0%
25%
50%
75%
0%
20%
40%
60%
E
CM
Stem-like
EM
other
MAIT-like
TEMRA
UMAP-2
UMAP-1
F
Stem-like
CM
EM
TEMRA
other
MAIT-like
Clusters:
CD45RO
CD45RA
CD62L
CD27
KLRG1
CD57
TCF7
CCR7
CD28
EOMES
ZEB2
ZBTB16
KLRB1
PRF1
GZMB
GZMK
GZMA
GZMH
GZMM
GNLY
CTSC
IFNG
TNF
IL2
Z-score: −3 +3
25%
50%
75%
G
CD4+: P= 0.23
C6, C8, C11, C12
C4
C1
C5, C9
C16, C19
Cluster proportions in S (%)
Cluster proportions in Z (%)
CD8+: P= 0.81
C9-14
C4
C8
Cluster proportions in S (%)
Cluster proportions in Z (%)

Figure S8, related to Fig. 4: VZV gE-specific T cells in Shingrix and Zostavax vaccine recipients include diverse subsets. (**A**) UMAP of VZV gE-reactive $CD4^+$ T cells of S and Z vaccine recipients. (**B**) Feature plots of $CD4^+$ T cell UCell gene scores supporting $CD4^+$ T cell subset annotation. (**C**) Bubble plot of CITE protein expression of classical T cell subset markers across $CD4^+$ T cell clusters. (**D**) Bubble plots of gene expression across $CD4^+$ T cell clusters showing subset-specific transcription factors (top), cytokines and effector molecules (bottom). The bubble size corresponds to the proportion of cells that express a given gene. (**E**) UMAP of VZV gE-reactive $CD8^+$ T cells of S and Z vaccine recipients. (**F**) Bubble plot of CITE protein expression (left) and gene expression data (middle and right) across $CD8^+$ T cell clusters showing classical T cell subset markers, transcription factors, cytokines and effector molecules. The bubble size corresponds to the proportion of cells that express a given gene. (**G**) Cluster distribution of $CD4^+$ or $CD8^+$ VZV gE-reactive T cells from Z and S vaccine recipients. Statistical analyses were performed comparing probability vectors and using permutation tests.

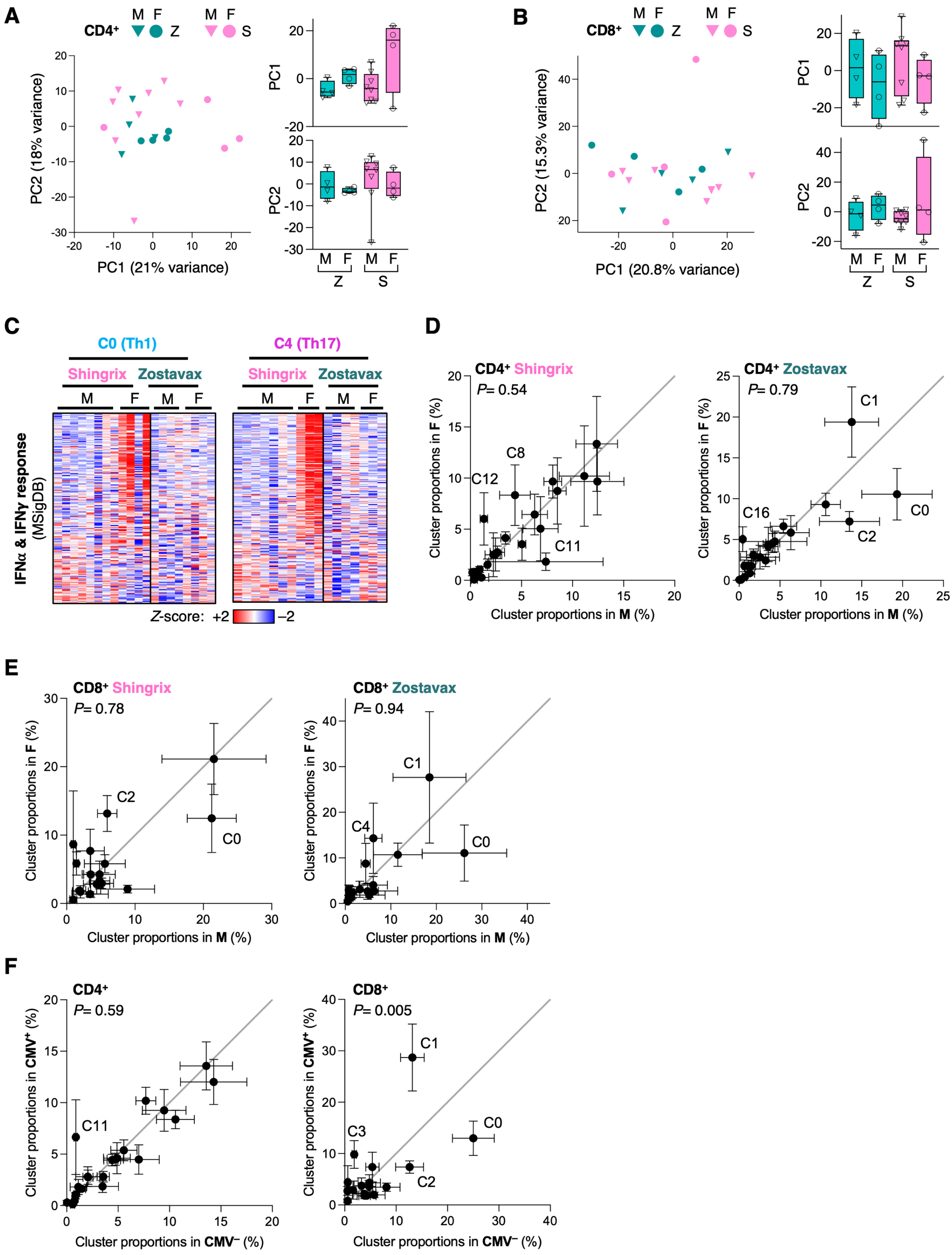
A
CD4+
M F
Z
M F
S
PC2 (18% variance)
PC1 (21% variance)
PC1
PC2
M F M F
Z S
B
CD8+
M F
Z
M F
S
PC2 (15.3% variance)
PC1 (20.8% variance)
PC1
PC2
M F M F
Z S
C
C0 (Th1)
C4 (Th17)
Shingrix
Zostavax
M F M F
IFNα & IFNγ response
(MSigDB)
Z-score: +2 −2
D
CD4+ Shingrix
P= 0.54
C8
C12
C11
CD4+ Zostavax
P= 0.79
C1
C16
C0
C2
Cluster proportions in F (%)
Cluster proportions in M (%)
E
CD8+ Shingrix
P= 0.78
C2
C0
CD8+ Zostavax
P= 0.94
C1
C4
C0
Cluster proportions in F (%)
Cluster proportions in M (%)
F
CD4+
P= 0.59
C11
CD8+
P= 0.005
C1
C3
C0
C2
Cluster proportions in CMV+ (%)
Cluster proportions in CMV− (%)

Figure S9, related to Fig. 4: Sex and CMV serostatus have minor contributions to the phenotypic differences in CD4$^+$ or CD8$^+$ VZV T cell response. (**A** and **B**) Principal component analysis on pseudo-bulk transcripts of VZV gE-reactive CD4$^+$ (A) and CD8$^+$ (B) T cells from Z and S vaccine recipients (left). PC-1 and 2 are shown as box plots (right). (**C**) Gene expression heatmaps for gene sets "IFNα response" plus "IFN**γ** response" (MSigDB) based on pseudo-bulk expression comparing S versus Z vaccine recipients for CD4$^+$ T cell clusters C0 (Th1) and C4 (Th17). (**D**) Relative cluster frequencies of CD4$^+$ VZV gE-reactive T cells in Shingrix and Zostavax vaccine recipients comparing female (F) and male (M) vaccine recipients. (**E**) As in (D) but for CD8$^+$ VZV gE-reactive T cells. (**F**) Relative cluster frequencies of CD4$^+$ or CD8$^+$ VZV gE-reactive T cells comparing individuals with CMV positive serostatus and those with CMV negative serostatus. Data show median (A and B) or mean ± SEM (D to F). All datapoints represent distinct biological replicates. Statistical analyses were performed comparing probability vectors and permutation tests (D, E, and F).

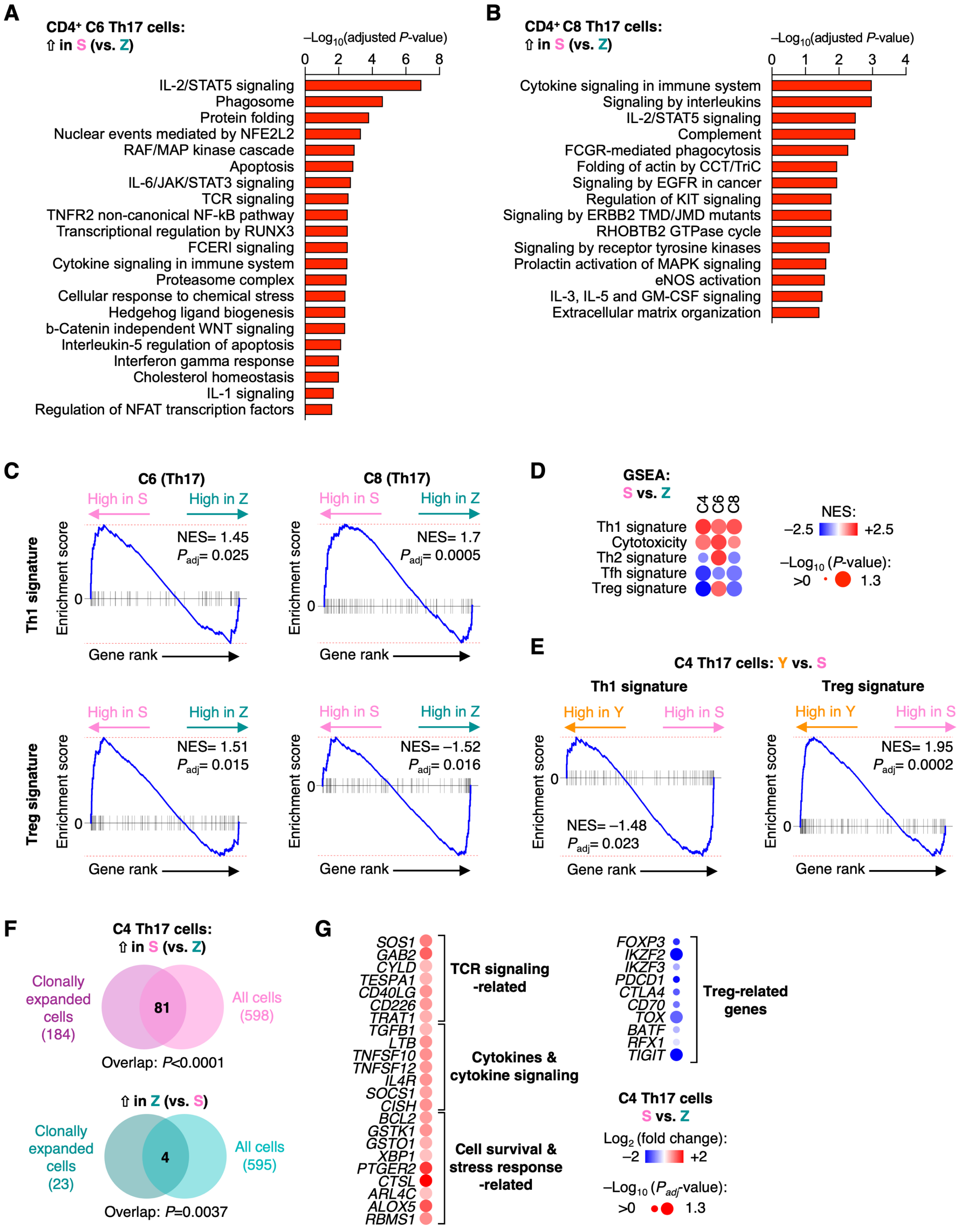

Figure S10, related to Fig. 5: VZV gE-reactive CD4$^+$ T cells in all Th17-related clusters share gene expression profiles indicating increased functionality in Shingrix recipients. (**A** and

B) Pathway enrichment for CD4$^+$ clusters C6 (A) and C8 (B) of DEGs which were higher expressed in S than in Z. (**C**) GSEA of C6 and C8 pseudo-bulk gene expression of Z versus S for concordance with Th1 and Treg gene sets. NES, normalized enrichment score. (**D**) As in (C) but summarizing GSEA for different gene sets and Th17 clusters. (**E**) As in (C) but for pseudo-bulk gene expression of young Varivax vaccine recipients discussed in Figs. 1 to 3 (Y) versus S. (**F**) Gene expression analysis of clonally expanded (2+ cells per TCR sequence) C4 Th17 cells comparing Z versus S using single cell differential analysis. Venn diagram shows the overlap of DEGs with the initial pseudo-bulk analysis of all C4 Th17 cells in Z versus S samples (Fig. 5A). The overlap of differential genes in both differential approaches was significant as assessed by Chi-square tests. (**G**) Heatmap based on clonally expanded Th17 cell gene expression analyses as in (F) showing selected genes found in Fig. 5C and 5E.

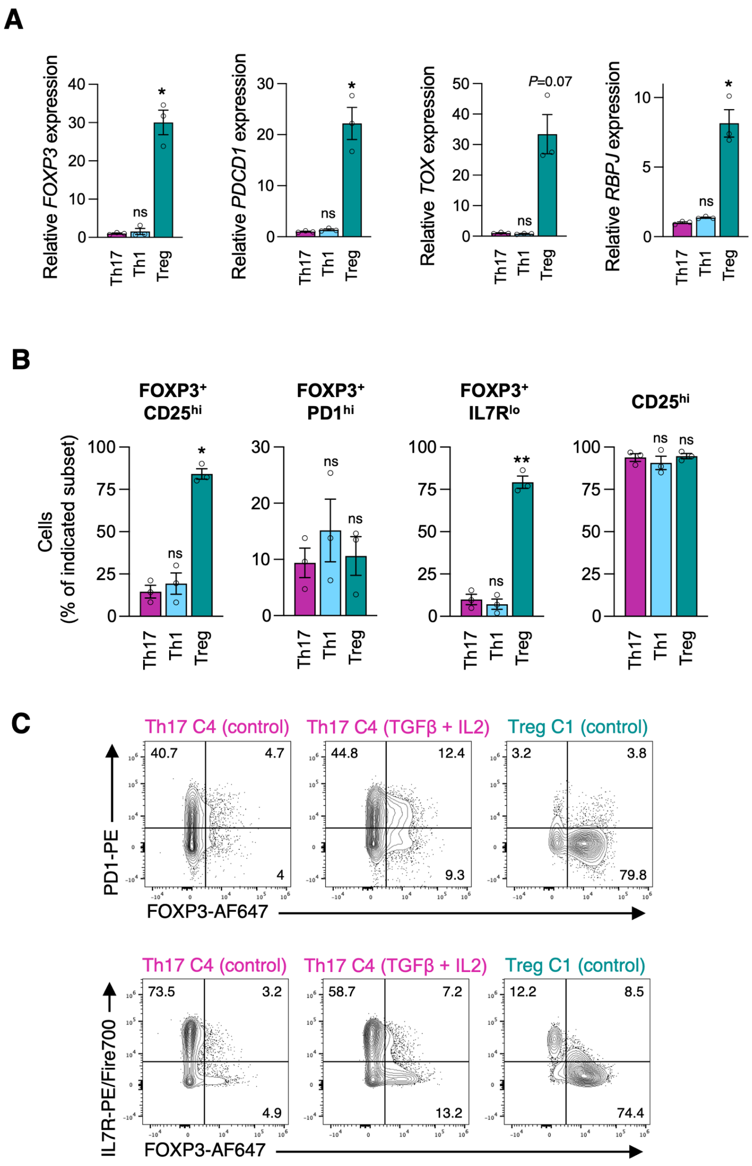

Figure S11, related to Fig. 6: Induction of C1 Treg-like signatures in C4 Th17 cells. (**A**) qPCR quantitative analysis of cells with a C4 Th17, C0 Th1 or C1 Treg phenotype that were FACS-collected from peripheral memory CD4$^+$ T cells of older adults and stimulated with αCD3/αCD28 antibodies for 3 days. (**B**) Flow cytometry quantitation of cells as in (A) but stimulated for 7 days. (**C**) Flow cytometry plots of Th17 or Treg cells as in (A) that were stimulated with αCD3/αCD28 in the absence of presence of 25 ng/ml TGFβ and 500 U/ml IL2 for 7 days. Data show mean ± SEM (A and B). All datapoints represent distinct biological replicates and data from one representative

experiment is shown. Data were compared by one-way ANOVA with Šídák's multiple comparisons test (A and B). *P<0.05, **P<0.01. ns, not significant.

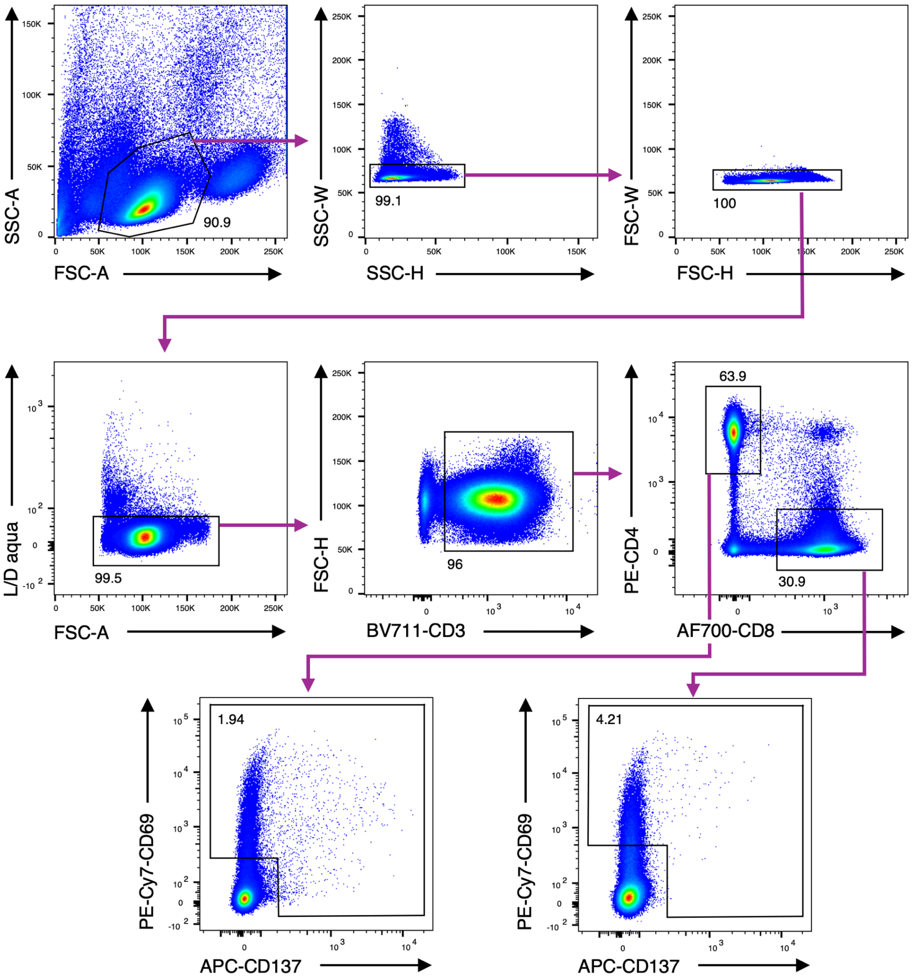

Figure S12: FACS gating strategy to collect VZV gE-reactive T cells for single cell sequencing.

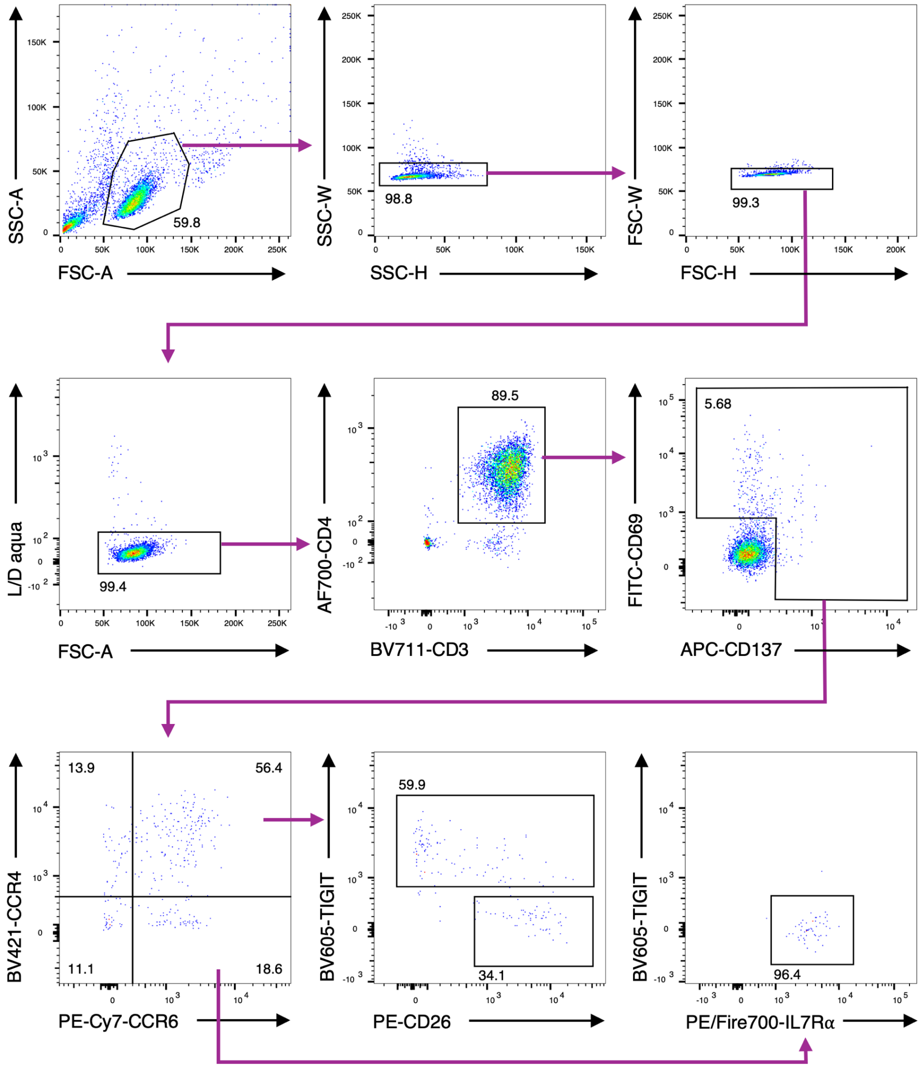

Figure S13: FACS gating strategy to collect phenotypic subsets of VZV gE-reactive T cells for functional studies.

Table S1: Patient demographics for single cell sequencing samples

Volunteers for single cell sequencing	30
Female	46.7%
Male	53.3%
White	83.3%
Asian	13.3%
American Indian/Alaska Native	3.3%
Race Unknown / Not Reported	3.3%
Not Hispanic or Latino	96.6%
Ethnicity Unknown / Not Reported	3.3%

	Varivax, Young	Zostavax, Older	Shingrix, Older
Volunteers	9	8	13
Age at sample collection (years)	24.3 ± 2.9	75.5 ± 7.3	69.5 ± 7.3
Age at last VZV vaccination (years)	9.8 ± 3.3	69.5 ± 7.6	65.6 ± 7.4
Years since last vaccination (years)	14.4 ± 1.1	6 ± 0.8	3.4 ± 0.5

Table S2: Patient co-morbidities and key medication for single cell sequencing samples

Volunteers for single cell sequencing	Zostavax	Shingrix
Total number	8	13
Gender (M/F)	4/4	8/5
Major comorbidities	3 (1-4)	3 (1-5)
Diabetes mellitus	3 (38%)	3 (23%)
Atrial fibrillation	2 (25%)	6 (46%)
Chronic kidney disease	1 (13%)	3 (23%)
Coronary artery disease	1 (13%)	2 (15%)
Hypertension	3 (38%)	5 (38%)
Hyperlipidemia	5 (63%)	9 (69%)
Asthma	1 (13%)	3 (23%)
COPD	2 (25%)	0 (0%)
Medication		
Statin	4 (50%)	8 (62%)
Anti-diabetics	2 (25%)	3 (23%)

Supplementary auxiliary files:

Table S3 (Excel): Sample information for single cell sequencing.

Table S4 (Excel): Antibodies for flow cytometry.

Table S5 (Excel): TotalSeq-B antibodies for CITE-seq.

Table S6 (Excel): Pseudo-bulk differential gene expression results of VZV-reactive T cells comparing Y and O groups.

Table S7 (Excel): Pseudo-bulk differential gene expression results of VZV-reactive T cells comparing S and Z groups.

Table S8 (Excel): Gene sets for enrichment analyses.
